# Supplementary material for: Retrospective review of the management of acute infections and the indications for antibiotic prescription in primary care in northern Thailand
Source: BMJ Open. 2018 Jul 30;8(7):e022250. doi: 10.1136/bmjopen-2018-022250 (PMC6067334; doi:10.1136/bmjopen-2018-022250)
Supplement: Supplementary data [file bmjopen-2018-022250supp001.pdf]

## Supplementary material

Antibiotic search list

- Amoxicillin
- Cefixime
- Ceftriaxone
- Cephalexin
- Ciprofloxacin
- Co-amoxiclav/ augmentin
- Co-trimoxazole/bactrim
- Dicloxacillin
- Doxycycline
- Erythromycin
- Metronidazole
- Norfloxacin
- Penicillin V
- Roxithromycin
- TC mycin/ tetracycline

*Table S1: ICD 10 codes for infection used for the inclusion criteria*

| Code | Description | Excluded code (number) |
|---|---|---|
| **A00-B99** | Certain infectious and parasitic diseases | A15 (167), A16 (29), A18 (7), A31.9 (1), B18 (18), B24 (85), B85 (671) |
| **G00-G07** | Inflammatory diseases of the central nervous system | |
| **H00-01** | Hordeolum, chalazion and other inflammation of the eyelid | H01.1 (35) |
| **H05.0** | Acute inflammation of orbit | |
| **H10** | Conjunctivitis | |
| **H60-H70** | Otitis externa, otitis media and mastoiditis | H61 (112) |
| **H72-73** | Perforation and other disorders of the tympanic membrane | H73.9 (2) |
| **J00-43** | Respiratory tract infections | J30 (150), J31 (8), J33 (1), J35.1 (1) |
| **J47** | Bronchiectasis | |
| **K05** | Gingivitis and periodontal diseases | Exclude all (9,469) |
| **K11-12** | Diseases of salivary glands, stomatitis and related lesions | K11.1 (3), K11.88 (2), K11.9 (1), K12.0 (682), K12.1 (716) |
| **K35-37** | Appendicitis | |
| **K57** | Diverticulitis | K57 (2) |

| | | |
|---|---|---|
| **K61** | Abscess of anal and rectal regions | |
| **K81** | Cholecystitis | |
| **K83-85** | Cholangitis and pancreatitis | |
| **L00-08** | Infections of the skin and subcutaneous tissue | |
| **L20-22** | Dermatitis | L20 (23), L21 (19), L22 (5) |
| **L30.3** | Infective dermatitis | |
| **L70-73.2** | Acne, rosacea follicular cysts and follicular disorders | |
| **M00-03** | Infectious arthropathies | M0013 (1), M0023 (1), M0167 (1), M020 (1) |
| **M60** | Myositis | M60.1-M6099 (3,604) |
| **N10-11** | Tubulo-interstitial nephritis | |
| **N30** | Cystitis | |
| **N34** | Urethritis and urethral syndrome | |
| **N39.0** | Urinary tract infection, site not specified | |
| **N41** | Inflammatory diseases of prostate | |
| **N45** | Orchitis and epididymitis | |
| **N48-49** | Other disorders of male genital organs | N48.9 (1) |
| **N61** | Inflammatory disorders of breast | |
| **N70-76** | Inflammatory diseases of female pelvic organs | |
| **O08.0** | Genital tract and pelvic infection following abortion and ectopic and molar pregnancy | |
| **O23** | Infections of genitourinary tract in pregnancy | |
| **O85-86** | Puerperal sepsis and other puerperal infections | |
| **P35-9** | Infections specific to the perinatal period | |
| **R05** | Cough | |
| **R11** | Nausea and vomiting | |
| **R30** | Pain associated with micturition | |
| **R36** | Urethral discharge | |
| **R50** | Fever | |

*Table S2: The number of initial presentations for each inclusion criteria and the percentage prescribed antibiotics during their illness episode*

| Age (years) | Number of presentations n/N (%) | Number of patients receiving an antibiotic prescription n/N (%) |
|---|---|---|
| **0-4** | 18,073/83,659 (21.6) | 6,110/18,073 (33.8) |
| **5-11** | 13,775/83,659 (16.5) | 6,318/13,775 (45.9) |
| **12-24** | 10,533/83,659 (12.6) | 5,888/10,533 (55.9) |
| **25-39** | 11,025/83,659 (13.2) | 6,167/11,025 (55.9) |
| **40-64** | 23,134/83,659 (27.7) | 11,843/23,134 (51.2) |
| **65 or over** | 7,119/83,659 (8.5) | 2,915/7,119 (41) |
| **Total** | 83,659 (100) | 39,241/83,659 (46.9) |

*Table S3: The number of presentations per age group and the percentage of each group prescribed an antibiotic*

| Inclusion criteria | Total initial presentations | Antibiotic prescription during the illness episode |
|---|---|---|
| **History of fever n/N (%)** | 29,246/82,976 (35.3%) | 11,725/29,246 (40.1%) |
| **Temperature >37.5°C n/N (%)** | 10,508/76,644 (13.7%) | 5,003/10,508 (47.6%) |
| **ICD 10 code for infection n/N (%)** | 70,137/83,338 (84.2%) | 27,234/70,137 (38.8%) |
| **Antibiotic prescription n/N (%)** | 37,011/83,661 (44.2%) | 39,242/83,661 (46.9%) |

| Diagnosis | Number of presentations n/N (%) | Number of antibiotic prescriptions during the illness episode n/N (%) |
|---|---|---|
| **Respiratory** | | |
| Common cold | 34,549/53,819 (64.2) | 3,643/34,549 (10.5) |
| Acute sinusitis | 30/53,819 (0.1) | 25/30 (83.3) |
| Acute pharyngitis | 13,080/53,819 (24.3) | 11,607/13,080 (88.7) |
| Acute tonsillitis | 3,459/53,819 (6.4) | 3,014/3,459 (87.1) |
| Other URTIs | 357/53,819 (0.7) | 278/357 (77.9) |
| Acute LRTIs | 663/53,819 (1.2) | 541/663 (81.6) |
| Chronic bronchitis, emphysema & bronchiectasis | 60/53,819 (0.1) | 10/60 (16.7) |
| Cough | 1,621/53,819 (3) | 99/1,621 (6.1) |
| Sub total | 53,819 (100) | 19,217/53,819 (35.7) |
| **Gastrointestinal** | | |
| Bacterial intestinal infections or intoxications | 199/2,706 (7.4) | 127/199 (63.8) |
| Viral enteritis | 46/2,706 (1.7) | 4/46 (8.7) |
| GE & colitis | 2,412/2,706 (89.1) | 1,614/2,412 (66.9) |
| Appendicitis | 21/2,706 (0.8) | 2/21 (9.5) |
| Other | 9/2,706 (0.3) | 2/9 (22.2) |
| Sialoadenitis | 19/2,706 (0.7) | 16/19 (84.2) |
| Sub total | 2,706 (100) | 1,765/2,706 (65.2) |
| **Skin** | | |
| Infective dermatitis | 85/4,060 (2.1) | 70/85 (82.4) |
| Dermatophytosis | 902/4,060 (22.2) | 92/902 (10.2) |
| Other superficial mycoses | 197/4,060 (4.9) | 14/197 (7.1) |
| Candidiasis | 101/4,060 (2.5) | 23/101 (22.8) |
| Other | 64/4,060 (1.6) | 52/64 (81.3) |
| Scabies & infestations | 52/4,060 (1.3) | 8/52 (15.4) |
| Cellulitis & abscesses | 841/4,060 (20.7) | 618/841 (73.5) |
| Bacterial skin infections | 533/4,060 (13.1) | 464/533 (87.1) |
| Furuncles, caruncles & cysts | 947/4,060 (23.3) | 780/947 (82.4) |
| Other local infection of the skin & subcutaneous tissue | 338/4,060 (8.3) | 290/338 (85.8) |
| Sub total | 4,060 (100) | 2,411/4060 (59.4) |
| **Eye** | | |
| Conjunctivitis | 2,097/2,698 (77.7) | 330/2,097 (15.7) |
| Hordeolum & chalazion | 319/2,698 (11.8) | 256/319 (80.3) |
| Other inflammation of the eyelid & orbit | 268/2,698 (9.9) | 98/268 (36.6) |
| Trachoma | 14/2,698 (0.5) | 5/14 (35.7) |

| | | |
|---|---|---|
| Sub total | 2,698 (100) | 689/2,698 (25.5) |
| **Ear** | | |
| Otitis externa | 464/753 (61.6) | 369/464 (79.5) |
| Otitis media | 243/753 (32.3) | 197/243 (81.1) |
| Mastoiditis | 16/753 (2.1) | 9/16 (56.3) |
| Perforation of tympanic membrane & other disorders | 30/753 (4) | 25/30 (83.3) |
| Sub total | 753 (100) | 600/753 (79.7) |
| **Urogenital** | | |
| Acute tubulo-interstitial nephritis | 36/1,871 (1.9) | 32/36 (88.9) |
| Other | 17/1,871 (0.9) | 12/17 (70.6) |
| Cystitis, UTI, dysuria, urethritis & urethral syndrome | 1,370/1,871 (73.2) | 1,291/1,370 (94.2) |
| Other disorders of male genital organs | 32/1,871 (1.7) | 20/32 (62.5) |
| Other inflammatory disorders of female pelvic organs | 148/1,871 (7.9) | 115/148 (77.7) |
| Other inflammatory disorders of the vagina & vulva | 268/1,871 (14.3) | 149/268 (55.6) |
| Sub total | 1,871 (100) | 1,619/1,871 (86.5) |
| **Other** | | |
| Bacterial | 85/3,208 (2.7) | 28/85 (32.9) |
| Unknown aetiology | 33/3,208 (1) | 14/33 (42.4) |
| Viral | 728/3,208 (22.7) | 153/728 (21) |
| Fungal | 36/3,208 (1.1) | 2/36 (5.6) |
| Protozoal | 10/3,208 (0.3) | 0/10 (0) |
| Parasitic | 1,880/3,208 (58.6) | 99/1,880 (5.3) |
| Nausea & vomiting | 268/3,208 (8.4) | 30/268 (11.2) |
| Fever of unknown or other origin | 168/3,208 (5.2) | 10/168 (6) |
| Sub total | 3,208 (100) | 336/3,208 (10.5) |

*Table S4: The number of presentations per diagnosis and system and whether antibiotics were prescribed for that illness episode*

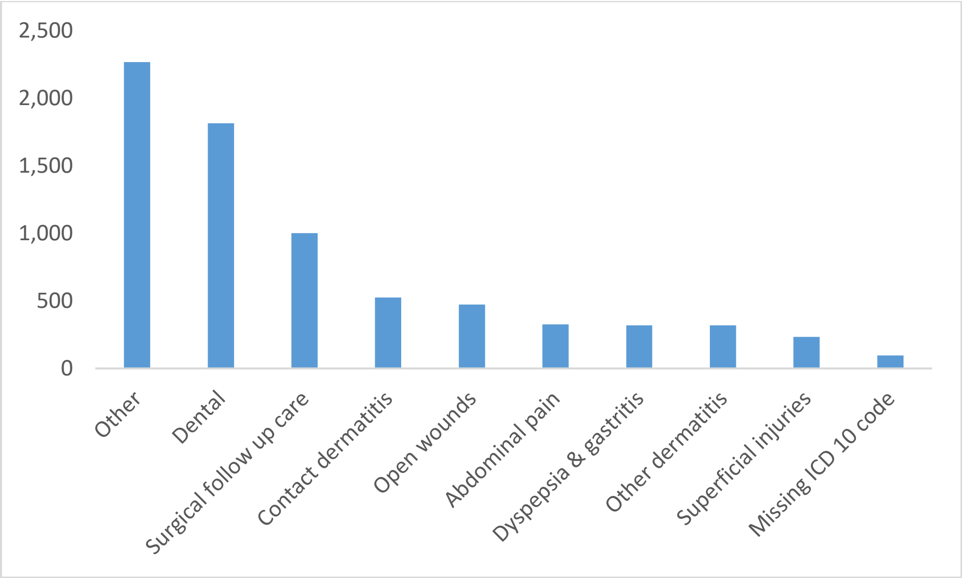


*Figure S1: Single diagnoses used for antibiotic prescriptions without a history of fever, temperature or ICD 10 code for infection*
