# Supplementary material for: Retrospective review of the management of acute infections and the indications for antibiotic prescription in primary care in northern Thailand
Source: BMJ Open. 2018 Jul 30;8(7):e022250. doi: 10.1136/bmjopen-2018-022250 (PMC6067334; doi:10.1136/bmjopen-2018-022250)
Supplement: Author's manuscript [file bmjopen-2018-022250.draft_revisions.pdf]

BMJ Open is committed to open peer review. As part of this commitment we make the peer review history of every article we publish publicly available.

When an article is published we post the peer reviewers' comments and the authors' responses online. We also post the versions of the paper that were used during peer review. These are the versions that the peer review comments apply to.

The versions of the paper that follow are the versions that were submitted during the peer review process. They are not the versions of record or the final published versions. They should not be cited or distributed as the published version of this manuscript.

BMJ Open is an open access journal and the full, final, typeset and author-corrected version of record of the manuscript is available on our site with no access controls, subscription charges or pay-per-view fees (http://bmjopen.bmj.com).

If you have any questions on BMJ Open's open peer review process please email info.bmjopen@bmj.com

BMJ Open

A Retrospective Survey of the Management of Acute Infections and the Indicators for Antibiotic Prescription in Primary Care in Northern Thailand

Journal:	*BMJ Open*
Manuscript ID	bmjopen-2018-022250
Article Type:	Research
Date Submitted by the Author:	09-Feb-2018

Complete List of Authors:	Greer, Rachel; Mahidol University, Mahidol-Oxford Tropical Medicine Research Unit, Faculty of Tropical Medicine; University of Oxford, Centre for Tropical Medicine and Global Health, Nuffield Department of Medicine,<br>Intralawan, Daranee; Chiangrai Regional Hospital, Social and Preventive Medicine Department<br>Mukaka, Mavuto; Mahidol University, Mahidol-Oxford Tropical Medicine Research Unit, Faculty of Tropical Medicine; University of Oxford, Centre for Tropical Medicine and Global Health, Nuffield Department of Medicine,<br>Wannapinij , Prapass; Mahidol University, Mahidol-Oxford Tropical Medicine Research Unit, Faculty of Tropical Medicine<br>Day, Nicholas; Mahidol University, Mahidol-Oxford Tropical Medicine Research Unit, Faculty of Tropical Medicine; University of Oxford, Centre for Tropical Medicine and Global Health, Nuffield Department of Medicine,<br>Nedsuwan, Supalert; Chiangrai Regional Hospital, Social and Preventive Medicine Department<br>Lubell, Yoel ; Mahidol University, Mahidol-Oxford Tropical Medicine Research Unit, Faculty of Tropical Medicine; University of Oxford, Centre for Tropical Medicine and Global Health, Nuffield Department of Medicine,

Keywords:	antibiotic use, PRIMARY CARE, Thailand, fever, Respiratory infections < THORACIC MEDICINE

SCHOLARONE™
Manuscripts

A RETROSPECTIVE SURVEY OF THE MANAGEMENT OF ACUTE INFECTIONS AND THE INDICATORS FOR ANTIBIOTIC PRESCRIPTION IN PRIMARY CARE IN NORTHERN THAILAND

Rachel C. Greer[1, 2], Daranee Intralawan[3], Mavuto Mukaka[1, 2], Prapass Wannapinij[1], Nicholas P.J. Day[1, 2], Supalert Nedsuwan[3], Yoel Lubell[1, 2]

[1] Mahidol-Oxford Tropical Medicine Research Unit, Faculty of Tropical Medicine, Mahidol University, Bangkok, Thailand
[2] Centre for Tropical Medicine and Global Health, Nuffield Department of Medicine, University of Oxford, Oxford, United Kingdom
[3] Social and Preventive Medicine Department, Chiangrai Regional Hospital, Chiangrai, Thailand

Corresponding author

Rachel Claire Greer. Address: Mahidol-Oxford Tropical Medicine Research Unit, Faculty of Tropical Medicine, Mahidol University, 3rd Floor, 60th Anniversary Chalermprakiat Building, 420/6 Rajvithi Road, Ratchathewi District, Bangkok 10400, Thailand. Email: rachel@tropmedres.ac Phone: +66830746941

Word count: 3344

For peer review only

ABSTRACT

Introduction Antibiotic use in low and middle income countries continues to rise despite the knowledge that antibiotic overuse can lead to antimicrobial resistance. There is a paucity of detailed data on the use of antibiotics in primary care in low resource settings.

Objective Describe the presentation of acute infections and the indicators for antibiotic prescription.

Design A two year retrospective survey.

Setting All 32 primary care units in one district in northern Thailand.

Participants Patients attending primary care with a history of fever, documented temperature, ICD 10 code for infection or prescribed a systemic antibiotic. Patients attending four centers where a study was initiated on C-reactive protein testing during this survey were excluded.

Outcome measures Antibiotic prescription rates, odds ratios for the indicators of antibiotic prescription and the clinical presentations.

Results 762,868 patients attended the health centers, of whom 103,196 met the inclusion criteria, 5,966 were excluded resulting in 97,230 attendances consisting of 83,661 illness episodes.

46.9% (39,242) of patients were prescribed an antibiotic during their illness. Indicators for antibiotic prescription in the multivariable logistic regression analysis included male sex (aOR 1.21 [CI 1.16-1.28], p <0.001), adults (aOR 1.77 [CI 1.57-2, p <0.001]) and a temperature >37.5°C (aOR 1.24 [CI 1.03-1.48, p 0.02]). 77.9% of the presentations were for respiratory related problems, of which 98.6% were upper respiratory tract infections. The leading infection diagnoses were common cold (50%), acute pharyngitis (18.9%) and acute tonsillitis (5%), which were prescribed antibiotics in 10.5%, 88.7% and 87.1% of cases respectively. Amoxicillin was the most commonly prescribed antibiotic.

Conclusions Nearly half of the patients received an antibiotic, the majority had a respiratory infection. The results can be used to plan interventions to improve the rationale use of antibiotics. Further studies in private facilities, pharmacies and dental clinics are required.

Word count: 295

Keywords: antibiotic use, primary care, Thailand, fever, respiratory infections

ARTICLE SUMMARY

Strengths and limitations of this study

- Over 80,000 illness episodes reviewed from all primary care units in a district, over a 2 year time period
- Wide range of infections included rather than focusing on one specific infection
- Use of routine electronic data (no Hawthorne Effect), making this work reproducible
- Only included public healthcare facilities
- Reliant on the correct coding and clinical diagnoses of illnesses

For peer review only

BACKGROUND

The proportion of global deaths attributable to communicable diseases has greatly reduced in recent years. Despite these improvements, 10.6% of deaths worldwide in 2015 were thought to be caused by lower respiratory tract infections (LRTIs), diarrhoea, and tuberculosis (TB).[1] In under 5 year olds, 51.8% of deaths worldwide were due to infectious causes in 2013, with pneumonia causing 14.8% of the deaths overall.[2] In Thailand in 2010, respiratory infections were the leading cause of hospitalisations and deaths of children under the age of 5.[3] Prompt access to appropriate antibiotics is vital to prevent many of these unnecessary deaths.[4] But while inappropriate or no treatment remains a clear cause for concern, global antibiotic consumption increased by 35% between 2000 and 2010, fueled by low and middle income countries (LMIC),[5] with the majority of antibiotics being consumed in the community.[6]

Overuse and misuse of antibiotics have been linked to the development of antimicrobial resistance (AMR).[6-8] Antibiotics prescribed to individuals in primary care have been associated with bacterial resistance in that individual for up to 12 months, and longer and more frequent antibiotic courses are more likely to cause resistance.[9] The World Health Organization have described AMR in Southeast Asia as being a 'burgeoning and often neglected' issue, stating that a 'post-antibiotic era' may become reality, resulting in common infections and minor injuries being untreatable.[10] In Thailand in 2010, there were an estimated 19,122 deaths attributable to multidrug resistant hospital-acquired infections.[11] In August 2016 the Thai government endorsed a national strategic plan for AMR which aims to optimize antimicrobial drug use and reduce the mean consumption of antimicrobials in humans by 20% by 2021.[12]

To appreciate the scale of the problem and to target future interventions a greater understanding of the acute infections presenting to primary care and the conditions for which antibiotics are used in LMICs is required. Such data, however, are limited,[13] with most studies deriving their estimates from a small sample of health providers and over a limited timeframe, therefore neglecting possible seasonal and spatial variation and other secular trends. In this paper we describe the indications for antibiotic prescription in 32 primary care units (PCUs) across a district in northern Thailand over a two year period.

METHOD

A retrospective computerised search of routinely collected data from primary care units in Mueang Chiangrai District between January 2015 and December 2016 was carried out.

Study sites

Thailand is an upper-middle income country. In 2016 its GDP (gross domestic product) was 406.8 billion (US$). The average life expectancy at birth is 75 years.[14] Chiangrai is the most northern province in Thailand and shares borders with Laos and Myanmar. It has a population of 1,157,302,[15] of whom 106,987 reside in Mueang Chiangrai District.[16]

Thailand's healthcare system is made up of public and private providers. Universal health coverage was established in 2002 following significant investment in the healthcare system and infrastructure since the 1970s. In rural and poorer areas primary healthcare is predominantly provided by the public healthcare system whereas in urban areas hospitals and private clinics play a larger role.[17] Antibiotics can be bought directly from pharmacies and local stores as well as being prescribed by healthcare workers.

For peer review only

In Mueang Chiangrai District family medicine doctors based at the provincial hospital oversee 32 public PCUs which are staffed primarily by two to five nurses and public health officers. On average PCUs look after 5000 patients each.[17] They provide care for acute and chronic conditions as well as providing preventative services such as immunisations, cervical screening and health education. Dental and traditional medicine services are also available. The furthest PCU is 2 hours' drive through the mountains from the provincial hospital in Chiangrai city (see figure 1). Finger-prick blood glucose tests are the only investigations routinely available on site.

Inclusion criteria

Patients were identified with at least one of the following:

- Systemic antibiotic prescription
- International Statistical Classification of Diseases (ICD) 10 code for infection (see supplementary material, table S1)
- Fever as the chief complaint
- Documented temperature >37.5°C at the PCU

We excluded patients attending PCUs used as study sites during or after a recent trial on the use of C-reactive protein (CRP) point of care tests (https://www.clinicaltrials.gov/ct2/show/NCT02758821?term=NCT02758821&rank=1).

Study outcomes

The primary outcome was the overall antibiotic prescription rate. Secondary outcomes included odds ratios for the indicators of antibiotic use, percentages of patients receiving antibiotics according to their diagnosis, percentages of individual antibiotics used and the frequency and type of acute infection presentations.

Data collection and statistical analysis

With the approval of the Chiangrai Provincial and Public Health Office (PHO), a research data manager accessed the PHO's routine medical records database to search for relevant patients and extract the pre-specified variables. Data collected included the PCU attended, patients' number, age, sex, date of visit, chief complaint, temperature, ICD 10 code, and drug prescriptions.

The study database was cleaned with the aid of two native northern Thai speaking study nurses. Our list of ICD codes for infection were reviewed with the other variables to ensure their appropriateness. Repeat attendances within one month were classed as one illness episode allowing for the detection of subsequent antibiotics or treatment changes. If no prescription was recorded we made the assumption that this was because no medication was given rather than the data being missing. All other indicators such as the chief complaint and temperature were taken from the initial presentation. Children were defined as being less than 12 years of age.

The ICD 10 codes were grouped into gastrointestinal, respiratory, skin, urogenital, eye, ear and other categories. Each category was further broken down into conditions such as acute sinusitis and acute pharyngitis. The respiratory category was also grouped into upper and lower respiratory tract infections.

For peer review only

The proportions of patients prescribed an antibiotic in different demographic groups were summarised and compared using the chi-square test. A logistic regression model was used to model the binary outcome of antibiotic prescription, both unadjusted and adjusted models were fitted. The odds ratios for the indicators of antibiotic prescription were first obtained from univariate logistic regression models and then considered in a multivariable model if they had a p value of <0.05. A temperature of >37.5°C was used rather than the more subjective history of fever. ICD codes were not included because of their strong association with antibiotic prescriptions (e.g. a health worker's diagnosis of acute pharyngitis and its affiliated ICD10 code was inherently associated with antibiotic prescription, as opposed to a diagnosis of 'common cold'). The PCUs were assumed to have a random effect in the model.

Monthly antibiotic prescriptions were weighted by the number of contributing PCUs per month and modelled over the two year period. We used a time-series analysis to separate long-term trends from seasonal variations .[18 19] Symmetric locally weighted moving averages were used. In this procedure, less weight was applied to time points (in months) furthest away from the present time point. A quarterly window was used to identify seasonality as follows: $\hat{X}_t = \frac{1}{9}(X_{t-2} + 2X_{t-1} + 3X_t + 2X_{t+1} + X_{t+2})$

Similarly a 12-month time-series window was used to obtain a trend line that would be sensitive to monthly changes but with reduced noise from seasonal variation:

$$\hat{X}_t = \frac{1}{24}(X_{t-6} + X_{t+6}) + \frac{1}{12}(X_t + X_{t-1} + X_{t+1} + X_{t-2} + X_{t+2} + X_{t-3} + X_{t+3} + X_{t-4} + X_{t+4} + X_{t-5} + X_{t+5})$$

Where $\hat{X}_t$ is the time-series modelled monthly prevalence of antibiotic prescription. Statistical significance was declared at alpha=0.05. Data analyses were performed with STATA version 14 (College Station, Texas, USA). Ethical approval was obtained from Chiangrai's Provincial and Public Health Office IRB (number 56/2560). Exemption was given by the Oxford Tropical Research Ethics Committee (OxTREC). Patients were not involved in the design of the study.

RESULTS

762,868 patients attended the PCUs between the 1st of January 2015 and the 31st of December 2016. The majority of patients' attendances included a chronic disease review or screening, the most common being screening for diseases such as diabetes, hypertension, mental health and dental disorders (145,410), essential hypertension reviews (98,822) and routine child health examinations (75,701).

The appropriateness of the ICD 10 codes for infection used in our inclusion criteria were reviewed alongside the other variables. For example, we found that patients with TB, HIV and Hepatitis B were only attending for regular medications rather than for acute illnesses so they were removed from the ICD 10 inclusion list. Mass head lice treatment at schools is carried out by the PCUs so these codes were also removed. The ICD 10 code 'K05' (dental) was also removed because it transpired that these patients are seen by dentists or dental nurses at the PCUs rather than by the regular PCU staff. All ICD 10 codes for myositis were removed from the inclusion criteria apart from M60.0 (infective myositis) because the other codes were being used for muscle pain or myalgia.

For peer review only

In total 103,196 attendances met our inclusion criteria; 5,966 were then excluded because the PCUs they attended were involved in the CRP study before or during their attendance, resulting in 97,230 attendances (12.8%) meeting our inclusion and exclusion criteria. 13,569 repeat attendances within one month were classed as a single illness episode, leaving 83,661 illness episodes.

Patient Characteristics

The median age was 24 years old with an interquartile range of 6 to 51 years old. Two patients had no age recorded. 54.7% of the patients were female (45,779) compared to 45.3% males (37,882) (p<0.001).

The proportion of patients meeting each inclusion criteria is shown in figure 2 and supplementary table S2. 29,246 (35.3%) patients presented with a history of fever, while 10,508 (13.7%) had a temperature of more than 37.5°C at presentation. 8,871 (11.6%) patients had both a history of fever and a temperature.

Antibiotics

Medications were prescribed for 81,691 (97.7%) illness episodes. 37,011 (44.2%) patients were prescribed an antibiotic during their first visit, and this increased to 39,242 (46.9%) throughout their illness episodes.

Antibiotics were prescribed to:

- 49.2% of men compared to 45% of women (p<0.001)
- 39% of children compared to 51.8% of adults (p <0.001)
- 40.1% of those with a history of fever
- 47.6% with a temperature >37.5°C
- 38.8% with an ICD 10 code for infection

The proportion of patients within each age group prescribed an antibiotic varied, with the lowest rates in young children (0-4 year olds, 33.8%), peaking in adults (12-39 year olds, 55.9%) and then diminishing in the elderly (aged 65 years and older, 41%, see supplementary material, table S3).

Indicators for antibiotic prescription in the multivariable logistic regression analysis were male sex (aOR 1.21 [CI 1.16-1.28], p <0.001), patients aged 12 years of age or older (compared to those less than 12 years old) (aOR 1.77 [CI 1.57-2, p <0.001]) and having a temperature of more than 37.5°C (aOR 1.24 [CI 1.03-1.48, p 0.02]).

Figure 3 is a time series plot for the monthly prevalence of antibiotic prescriptions. Overall there was no significant trend; incidence rate ratio (IRR) =0.99, 95% CI (0.990, 1.007), p=0.796, although there is a suggestion of a downward trend beginning in the final 6 months. The monthly prevalence of antibiotic prescriptions was at least 39% throughout the 2-year period. Overall prescription rates varied greatly between the PCUs from 8 to 71.6%, with prescribing consistently higher in adults than children.

The majority of patients prescribed an antibiotic received amoxicillin (56.7%) or dicloxacillin (25.1%). Other antibiotics prescribed include norfloxacin (8.9%), co-trimoxazole (4.2%), penicillin V (1.2%), roxithromycin (1.2%), metronidazole (1.2%), erythromycin 0.7%, cephalexin (0.4%) and tetracycline (0.2%).

For peer review only

Presentations and antibiotic prescriptions

The number of acute presentations with ICD 10 codes for infection related to a single system are shown in figure 4. 77.9% of these presentations were for respiratory related problems. 98.6% of these were diagnosed with an upper respiratory tract infection (URTI), 1.1% with an acute LRTI and 0.3% with a chronic LRTI, of these 36.1%, 81.8% and 53.5% were prescribed antibiotics respectively. The most common single infection diagnoses were common cold (34,549, 50%), acute pharyngitis (13,080, 18.9%) and acute tonsillitis (3,459, 5%), antibiotics were prescribed to 10.5%, 88.7% and 87.1% of the cases respectively (see table 1).

Diagnosis	Number of presentations n/N (%)	Episode antibiotics prescribed n/N (%)	Commonest antibiotic prescribed (%)
**Common cold**	34,549/69,115 (50)	3,643/34,549 (10.5)	Amoxicillin (71.7)
**Acute pharyngitis**	13,080/69,115 (18.9)	11,607/13,080 (88.7)	Amoxicillin (91.5)
**Acute tonsillitis**	3,459/69,115 (5)	3,014/3,459 (87.1)	Amoxicillin (93.4)
**Gastroenteritis & colitis unspecified**	2,412/69,115 (3.5)	1,614/2,412 (66.9)	Norfloxacin (68.8)
**Conjunctivitis**	2,097/69,115 (3.0)	330/2,097 (15.7)	Amoxicillin (56.4)
**Other helminthiases**	1,231/69,115 (1.8)	65/1,231 (5.3)	Amoxicillin (41.5)
**Cystitis**	1,230/69,115 (1.8)	1,165/1,230 (94.7)	Norfloxacin (75.9)

Table 1: Common diagnoses in patients with one single ICD 10 code for infection, whether antibiotics were prescribed and which antibiotic was mostly commonly used

Supplementary table S4 shows the number of individual infection diagnoses by systems and the rates of antibiotic prescriptions. Antibiotics were prescribed to 59.4% of skin infections, 81.1% of otitis media, 79.5% of otitis externa, 94.7% of cystitis, 80.3% of hordeolum (styes) and chalazions as well as 15.7% of conjunctivitis cases. Of the total antibiotics prescribed almost a third (29.6%) were given to those with acute pharyngitis, followed by common cold (9.3%), acute tonsillitis (7.7%), gastroenteritis & colitis (4.1%) and cystitis (3%) as the single infection diagnoses.

13.8% of patients (11,547) were prescribed antibiotics without a temperature, history of fever or ICD 10 code for infection. Of those who had a single ICD 10 code recorded 1,815 (24.6%) of these antibiotics were for dental reasons, 1,002 (13.6%) for surgical follow up care, 526 (7.1%) for contact dermatitis and 473 (6.4%) for open wounds, see supplementary material, figure S1. These patients were more likely to be male (54.3%, p value <0.001) and older (median age of 41 compared to 24 years old) than the main patient group. The distribution of ages and temperatures between the main patient group and those meeting only the antibiotic inclusion criteria were both significantly different (p value <0.001).

DISCUSSION

To the best of our knowledge this is the largest review of acute illness presentations and community antibiotic prescribing in a LMIC. Over a 2 year period there were more than 97,000 attendances to 32 PCUs for acute infections and nearly half these patients received an antibiotic, with no significant change in prescribing levels over the 2 year study period. Studies of this magnitude are required to

For peer review only

increase our knowledge of the scale of antibiotic prescribing as well as the common conditions they are used for.[20 21] Thailand's 2016 national strategic plan on AMR also highlighted the importance of monitoring and reporting antimicrobial consumption.[22]

Comparing overall antibiotic prescribing rates with other studies is challenging because of varying definitions of acute illnesses and the different patient populations. However the antibiotic prescribing rate in our study is more than double the prescribing in a Malaysian study but similar to studies in India and Laos.[23-25] A third of our patients had a history of fever, which is similar to a point prevalence study in India where fever was the most common symptom.[26] Almost 80% of the ICD 10 codes for infection were related to respiratory infections, which is consistent with respiratory infections being the leading cause of hospitalisations and deaths in Thai under 5 year olds[3] but is higher than the proportion of respiratory presentations in other South and Southeast Asian countries.[26 27] Antibiotic prescribing in Thailand for tonsillitis and pharyngitis remains high despite Group A beta-haemolytic Streptococci being isolated in only 3.8-7.9% of those with URTI.[28 29]

Thailand's Antibiotic Smart Use 2007 project targets three conditions which are unlikely to require antibiotic treatment but for which they are commonly prescribed; URTIs, acute diarrhoea and simple wounds.[30] In the first phase of this project overall antibiotic use in PCUs was reduced by between 39% and 46%. Prescriptions for the three target conditions reduced from 54.5% to 25.4%.[31] Despite the lower prescribing levels for common colds in our survey there were still 3,643 antibiotic prescriptions for this condition and 66.9% of those with gastroenteritis and colitis received antibiotics. Open wounds and superficial injuries were common diagnoses in those prescribed an antibiotic without a history of fever, temperature or ICD 10 code for infection. The results reveal the ongoing high levels of prescribing for these conditions and present an opportunity to further improve antibiotic use. Since October 2016 an antibiotic prescribing target of less than 20% for these three conditions has been incorporated into Thailand's rationale drug use service plan as well as the pay for performance health criteria, financial incentives are given to the PCUs achieving this target. A review of the long term effectiveness of this policy including any impact on patient safety is required.

Our study also identifies high levels of prescribing for skin infections, otitis media, otitis externa, cystitis, hordeolum (styes) and chalazions. A lack of available topical antibiotics may account for the high prescribing for skin infections as well as for otitis externa. However, despite antibacterial eye drops being available, 15.7% of conjunctivitis cases were still prescribed a systemic antibiotic. Urine dipstick tests are not available on site to assess patients with cystitis or suspected urinary tract infections. Introduction of these simple tests may help to rationalize prescribing for these conditions in a setting where urine cultures are not readily available or achievable.

While we did not set out to review dental prescribing, this area accounted for 25% of the antibiotics prescribed to those without a history of fever, temperature or ICD 10 code for infection, which warrants further investigation.

Some of the variation in antibiotic prescribing rates between PCUs may be accounted for by the degree of staff training. Two out of the three highest prescribing PCUs are staffed only by public health officers. The study findings are being used to guide educational updates and training for the PCU staff, with priority being given to those PCUs without nurses and with high prescription rates for conditions unlikely to require antibiotics.

For peer review only

A wide range of antibiotics are prescribed in the PCUs. Restrictions are in place for some broad-spectrum antibiotics such as amoxicillin and clavulanic acid (Co-amoxiclav) which cannot be prescribed. One area of concern is that less than 1% of the antibiotics being prescribed have activity against scrub typhus, which is the leading cause of hospital admission with acute undifferentiated fever in this region.[32]

Strengths and limitations

The main strength of this study is the large number of illness episodes included. The two year time period should allow for seasonal variations and disease epidemics. We reviewed prescribing in all of the PCUs in Mueang Chiangrai District which covers a large geographical area and has a range of rural and urban facilities, making the results generalizable to the region more broadly. Many studies have focused on prescribing for specific conditions such as URTIs but our study covers a wide range of infections that present in the community. Having research staff on site has been shown to influence healthcare workers' prescribing habits (the Hawthorne Effect), but due to the retrospective nature of the study this was not a source of bias. The use of routinely collected data means that this methodology could be repeated in other districts and provinces in Thailand, although a lot of the data are entered as free text which presents challenges for analysis. Searching for patients with a history of fever, for instance, was problematic because the Thai word 'ไข้' or fever is also part of the Thai words for patient, influenza, anti-pyrexials, etc.

Limitations of this study are that we only included public PCUs and have no data on antibiotic use by private clinics, pharmacies or family medicine doctors based at the provincial hospital, which requires further study. The PCU data is taken from routine electronic records and in some instances there were tranches of missing data (5 PCUs had missing data for several months). Verifying the quality of the data is also challenging; coding of clinical diagnoses for instance using ICD 10 could be inaccurate or inconsistent between healthcare workers. Our decision to class all attendances within a one month period as a single illness episode means that we may have incorrectly classed some new illnesses as a repeat attendance but did allow us to review antibiotic prescribing over the course of the illness.

CONCLUSIONS

This study provides much needed insight into the use of antibiotics in primary care in northern Thailand, allowing targeting of interventions to improve the rationale use of antibiotics. Nearly half of all patients attending with an acute illness received an antibiotic. The majority of presentations were for respiratory infections. Further education and resources are required to support clinicians in the targeting of antibiotics. This could include the introduction of clinical algorithms and point of care tests such as CRP and urine dipsticks. Antibiotic guidelines are required for common conditions seen in primary care outside of the current Antibiotic Smart Use policy. Further studies including qualitative work are required to appreciate the use of antibiotics in other settings such as private facilities, pharmacies and dental clinics.

For peer review only

Funding statement

This research received no specific grant from any funding agency in the public, commercial or not-for-profit sectors. This study was part of the Wellcome Trust Major Overseas Programme in SE Asia (grant number 106698/Z/14/Z).

Competing interests

None declared

Author contribution statement

All authors (RCG, DI, MM, PW, NPJD, SN and YL) were involved in the design of the study. PW collected the data. RCG carried out the analysis with support from YL. MM provided statistical support. RCG, YL, DI SN and MM interpreted the data. RCG wrote the first draft of the paper. YL, NPJD and MM reviewed subsequent drafts. All authors (RCG, DI, MM, PW, NPJD, SN and YL) contributed to and approved the final draft for publication.

Acknowledgements

We would like to thank Wasuphal Kanjana for his help with data collection, Nipaphan Kanthawang and Pratakpong Wongkiti for their help with data cleaning and Areerat Thaiprakhong for her assistance with figure 1.

Data sharing statement

We are unable to share additional unpublished data which falls under the jurisdiction of the Chiang Rai PHO.

Ethical approval

Ethical approval was obtained from Chiangrai's Provincial and Public Health Office IRB (number 56/2560). Exemption was given by the Oxford Tropical Research Ethics Committee (OxTREC). Individual informed consent was not required.

For peer review only

REFERENCES

1. The World Health Organization (WHO). The top 10 causes of death. 2017 [Available from: http://www.who.int/mediacentre/factsheets/fs310/en/] (Accessed January 2018)
2. Liu L, Oza S, Hogan D, et al. Global, regional, and national causes of child mortality in 2000-13, with projections to inform post-2015 priorities: an updated systematic analysis. *Lancet* 2015;385(9966):430-40. doi: 10.1016/S0140-6736(14)61698-6 [published Online First: 2014/10/05]
3. Teeratakulpisarn J, Uppala R, Thepsuthammarat K, et al. Burden of Acute Lower Respiratory Infection in Children in Thailand in 2010: Have We Achieved the National Target in Under-five Morbidity and Mortality? *Journal of the Medical Association of Thailand = Chotmaihet thangphaet* 2012;95(7):S87-96.
4. Laxminarayan R, Matsoso P, Pant S, et al. Access to effective antimicrobials: a worldwide challenge. *Lancet* doi: 10.1016/S0140-6736(15)00474-2
5. Van Boeckel TP, Gandra S, Ashok A, et al. Global antibiotic consumption 2000 to 2010: an analysis of national pharmaceutical sales data. *Lancet Infect Dis*;14(8):742-50. doi: 10.1016/S1473-3099(14)70780-7
6. The European Centre for Disease Prevention and Control (ECDC). Surveillance of antimicrobial consumption in Europe 2012. 2014 [Available from: https://ecdc.europa.eu/sites/portal/files/media/en/publications/Publications/antimicrobial-consumption-europe-esac-net-2012.pdf.] (Accessed August 2017)
7. Laxminarayan R, Duse A, Wattal C, et al. Antibiotic resistance - the need for global solutions. *Lancet Infect Dis* 2013;13(12):1057-98. doi: 10.1016/S1473-3099(13)70318-9
8. Gelband H, Miller-Petrie M, Pant S, et al. The State of the World's Antibiotics: Center for Disease Dynamocs, Ecomomics & Policy (CDDEP). 2015 [Available from: https://www.cddep.org/publications/state_worlds_antibiotics_2015/] (Accessed October 2017)
9. Costelloe C, Metcalfe C, Lovering A, et al. Effect of antibiotic prescribing in primary care on antimicrobial resistance in individual patients: systematic review and meta-analysis. *BMJ* 2010;340 doi: 10.1136/bmj.c2096
10. The World Health Organization (WHO). Antimicrobial Resistance. Global Report on Surveillance 2014 [Available from: http://apps.who.int/iris/bitstream/10665/112642/1/9789241564748_eng.pdf?ua=1] (Accessed March 2017)
11. Lim C, Takahashi E, Hongsuwan M, et al. Epidemiology and burden of multidrug-resistant bacterial infection in a developing country. *eLife* 2016;5:e18082. doi: 10.7554/eLife.18082
12. Tangcharoensathien V, Sattayawutthipong W, Kanjanapimai S, et al. Antimicrobial resistance: from global agenda to national strategic plan, Thailand. *Bulletin of the World Health Organization* 2017;95(8):599-603. doi: 10.2471/BLT.16.179648
13. Zellweger RM, Carrique-Mas J, Limmathurotsakul D, et al. A current perspective on antimicrobial resistance in Southeast Asia. *J Antimicrob Chemother* 2017;72(11):2963-72. doi: 10.1093/jac/dkx260
14. The World Bank Group. Thailand data 2017 [Available from: https://data.worldbank.org/country/thailand] (Accessed October 2017)
15. National Statistical Office (NSO). Preliminary Report The 2010 Population and Housing Census (Whole Kingdom). Bangkok, 2011. [Available from http://popcensus.nso.go.th/upload/popcensus-08-08-55-E.pdf] (Accessed August 2017)

For peer review only

16. Department of Provincial Administration. Official Statistics Registration Systems, Thailand, 2016. [Available from: http://stat.dopa.go.th/stat/statnew/upstat_age.php] (Accessed November 2017)
17. Asia Pacific Observatory on Health Systems and Policies. The Kingdom of Thailand Health System Review. *Health Syst Transit* 2015;5(5)
18. Moineddin R, Upshur RE, Crighton E, et al. Autoregression as a means of assessing the strength of seasonality in a time series. *Popul Health Metr* 2003;1(1):10. doi: 10.1186/1478-7954-1-10 [published Online First: 2003/12/17]
19. Wall EC, Everett DB, Mukaka M, et al. Bacterial Meningitis in Malawian Adults, Adolescents, and Children During the Era of Antiretroviral Scale-up and Haemophilus influenzae Type b Vaccination, 2000–2012. *Clin Infect Dis* 2014;58(10):e137-e45. doi: 10.1093/cid/ciu057
20. O'Brien K, Bellis TW, Kelson M, et al. Clinical predictors of antibiotic prescribing for acutely ill children in primary care: an observational study. *Br J Gen Pract* 2015;65(638):e585.
21. Petersen I, Hayward AC. Antibacterial prescribing in primary care. *J Antimicrob Chemother* 2007;60(suppl_1):i43-i47. doi: 10.1093/jac/dkm156
22. Sumpradit N, Wongkongkathep S, Poonpolsup S, et al. New chapter in tackling antimicrobial resistance in Thailand. *BMJ* 2017;358
23. Keohavong B, Syhakhang L, Sengaloundeth S, et al. Rational use of drugs: prescribing and dispensing practices at public health facilities in Lao PDR. *Pharmacoepidemiol Drug Saf* 2006;15(5):344-47. doi: 10.1002/pds.1169
24. Ab Rahman N, Teng CL, Sivasampu S. Antibiotic prescribing in public and private practice: a cross-sectional study in primary care clinics in Malaysia. *BMC Infect Dis* 2016;16 doi: 10.1186/s12879-016-1530-2
25. Kotwani A, Holloway K. Trends in antibiotic use among outpatients in New Delhi, India. *BMC Infect Dis* 2011;11:99-99. doi: 10.1186/1471-2334-11-99
26. Salvi S, Apte K, Madas S, et al. Symptoms and medical conditions in 204912 patients visiting primary health-care practitioners in India: a 1-day point prevalence study (the POSEIDON study). *Lancet Glob Health* 2015;3(12):e776-e84. doi: 10.1016/S2214-109X(15)00152-7
27. Mimi O, Tong SF, Nordin S, et al. A Comparison of Morbidity Patterns in Public and Private Primary Care Clinics in Malaysia. *Malaysian Family Physician : the Official Journal of the Academy of Family Physicians of Malaysia* 2011;6(1):19-25.
28. Boonyasiri A, Thamlikitkul V. Effectiveness of multifaceted interventions on rational use of antibiotics for patients with upper respiratory tract infections and acute diarrhea. *Journal of the Medical Association of Thailand = Chotmaihet thangphaet* 2014;97(3 SUPPL. 3):S13-S19.
29. Treebupachatsakul P, Tiengrim S, Thamlikitkul V. Upper respiratory tract infection in Thai adults: prevalence and prediction of bacterial causes, and effectiveness of using clinical practice guidelines. *Journal of the Medical Association of Thailand = Chotmaihet thangphaet* 2006;89(8):1178-86.
30. Sumpradit N, Chongtrakul P, Anuwong K, et al. Antibiotics Smart Use: a workable model for promoting the rational use of medicines in Thailand. *Bulletin of the World Health Organization* 2012;90(12):905-13. doi: 10.2471/BLT.12.105445
31. The World Health Organization (WHO). The Pursuit of Responsible Use of Medicine: Sharing and Learning from Country Experiences. 2012 [Available from:http://www.who.int/medicines/publications/responsible_use/en/] (Accessed December 2017)
32. Wangrangsimakul T, Althaus T, Mukaka M, et al. Causes of acute undifferentiated fever and the utility of biomarkers in Chiangrai, northern Thailand. Submitted

For peer review only

Figure 1: Chiangrai and the 32 PCUs

Figure 2: A Venn diagram to show the inclusion criteria

Figure 3: Trend and seasonality of antibiotic prescriptions overlaid by mean antibiotic prescription rates per PCU

Figure 4: Number of acute presentations by single systems and whether antibiotics were prescribed

For peer review only

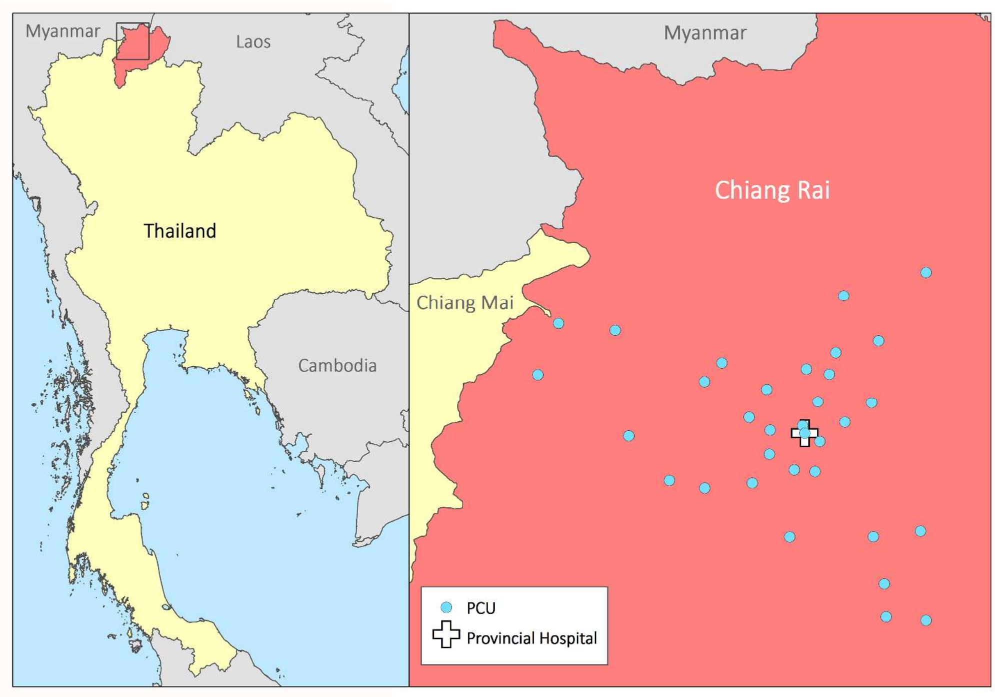

Figure 1: Chiangrai and the 32 PCUs

354x246mm (300 x 300 DPI)

For peer review only

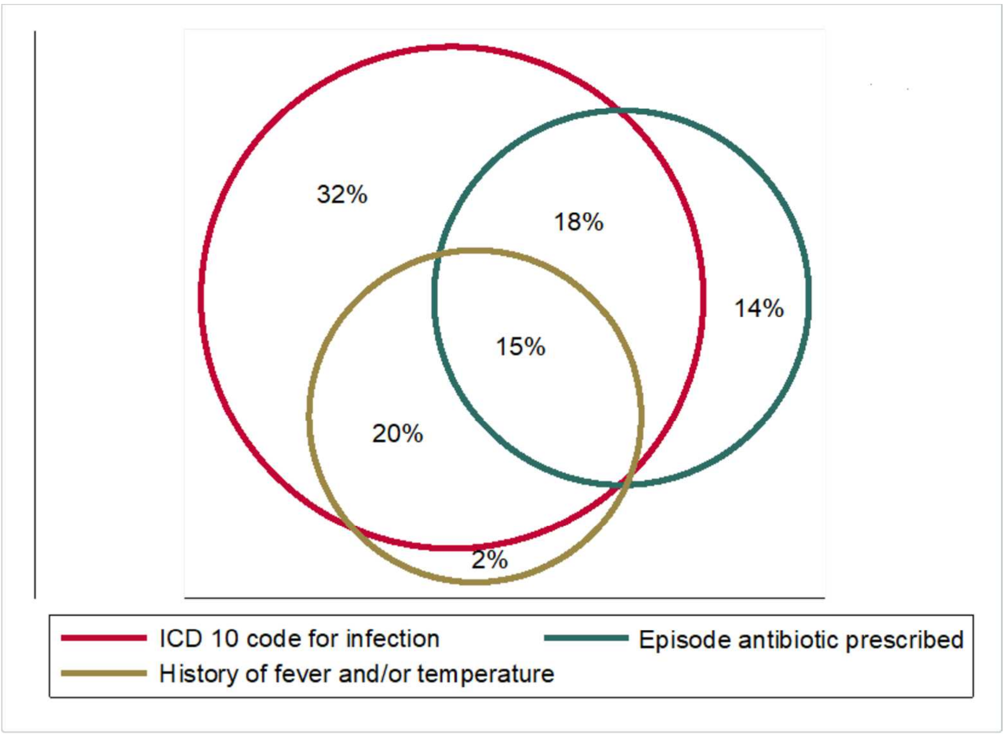

Figure 2: a Venn diagram to show the inclusion criteria

264x192mm (144 x 144 DPI)

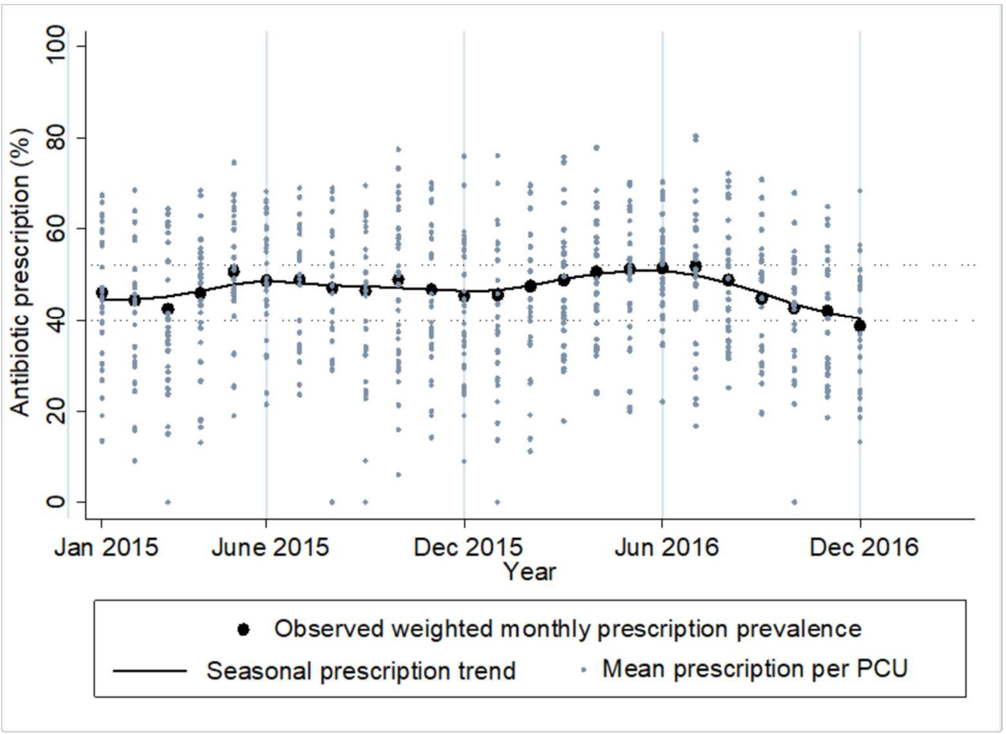

Figure 3: Trend and seasonality of antibiotic prescriptions overlaid by mean antibiotic prescription rates per PCU

127x92mm (300 x 300 DPI)

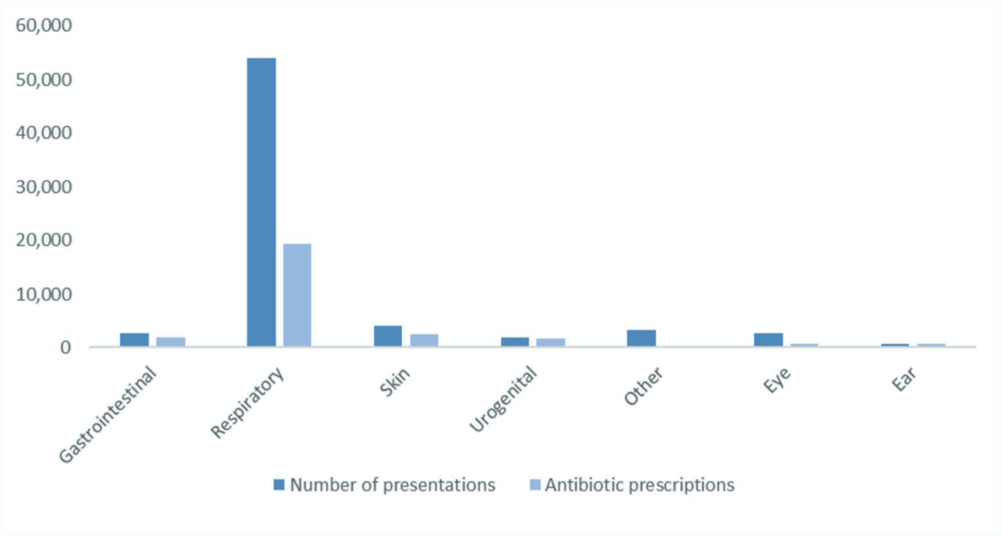

Figure 4: Number of acute presentations by single systems and whether antibiotics were prescribed

264x141mm (144 x 144 DPI)

review only

SUPPLEMENTARY MATERIAL

Code	Description	Excluded
**A00-B99**	Certain infectious and parasitic diseases	A15, A16, A18, A319, B18, B24, B85
**G00-G07**	Inflammatory diseases of the central nervous system	
**H00-01**	Hordeolum, chalazion and other inflammation of the eyelid	H01.1
**H05.0**	Acute inflammation of orbit	
**H10**	Conjunctivitis	
**H60-H70**	Otitis externa, otitis media and mastoiditis	H61
**H72-73**	Perforation and other disorders of the tympanic membrane	H73.9
**J00-43**	Respiratory tract infections	J30, J31, J33, J35.1
**J47**	Bronchiectasis	
**K05**	Gingivitis and periodontal diseases	Exclude all
**K11-12**	Diseases of salivary glands, stomatitis and related lesions	K11.1, K11.88, K11.9, K12.0, K12.1
**K35-37**	Appendicitis	
**K57**	Diverticulitis	K57
**K61**	Abscess of anal and rectal regions	
**K81**	Cholecystitis	
**K83-85**	Cholangitis and pancreatitis	
**L00-08**	Infections of the skin and subcutaneous tissue	
**L20-22**	Dermatitis	Exclude all
**L30.3**	Infective dermatitis	
**L70-73.2**	Acne, rosacea follicular cysts and follicular disorders	
**M00-03**	Infectious arthropathies	M0013, M0023, M0167, M020
**M60**	Myositis	M60.1-M6099
**N10-11**	Tubulo-interstitial nephritis	
**N30**	Cystitis	
**N34**	Urethritis and urethral syndrome	
**N39.0**	Urinary tract infection, site not specified	
**N41**	Inflammatory diseases of prostate	
**N45**	Orchitis and epididymitis	
**N48-49**	Other disorders of male genital organs	N48.9
**N61**	Inflammatory disorders of breast	
**N70-76**	Inflammatory diseases of female pelvic organs	

For peer review only

**O08.0**	Genital tract and pelvic infection following abortion and ectopic and molar pregnancy
**O23**	Infections of genitourinary tract in pregnancy
**O85-86**	Puerperal sepsis and other puerperal infections
**P35-9**	Infections specific to the perinatal period
**R05**	Cough
**R11**	Nausea and vomiting
**R30**	Pain associated with micturition
**R36**	Urethral discharge
**R50**	Fever

Table S1: ICD 10 codes for infection used for the inclusion criteria

Inclusion criteria	Total initial presentations	Antibiotic prescription during the illness episode
**History of fever n/N (%)**	29,246/82,976 (35.3%)	11,725/29,246 (40.1%)
**Temperature >37.5°C n/N (%)**	10,508/76,644 (13.7%)	5,003/10,508 (47.6%)
**ICD 10 code for infection n/N (%)**	70,137/83,338 (84.2%)	27,234/70,137 (38.8%)
**Antibiotic prescription n/N (%)**	37,011/83,661 (44.2%)	39,242/83,661 (46.9%)

Table S2: the number of initial presentations for each inclusion criteria and the percentage prescribed antibiotics during their illness episode

Age (years)	Number of presentations n/N (%)	Number of patients receiving an antibiotic prescription n/N (%)
**0-4**	18,073/83,659 (21.6)	6,110/18,073 (33.8)
**5-11**	13,775/83,659 (16.5)	6,318/13,775 (45.9)
**12-24**	10,533/83,659 (12.6)	5,888/10,533 (55.9)
**25-39**	11,025/83,659 (13.2)	6,167/11,025 (55.9)
**40-64**	23,134/83,659 (27.7)	11,843/23,134 (51.2)
**65 or over**	7,119/83,659 (8.5)	2,915/7,119 (41)
**Total**	83,659 (100)	39,241/83,659 (46.9)

Table S3: the number of presentations per age group and the percentage of each group prescribed an antibiotic

Diagnosis	Number of presentations n/N (%)	Number of antibiotic prescriptions during the illness episode n/N (%)
**Respiratory**		
Common cold	34,549/53,819 (64.2)	3,643/34,549 (10.5)
Acute sinusitis	30/53,819 (0.1)	25/30 (83.3)
Acute pharyngitis	13,080/53,819 (24.3)	11,607/13,080 (88.7)
Acute tonsillitis	3,459/53,819 (6.4)	3,014/3,459 (87.1)
Other URTIs	357/53,819 (0.7)	278/357 (77.9)
Acute LRTIs	663/53,819 (1.2)	541/663 (81.6)
Chronic bronchitis, emphysema & bronchiectasis	60/53,819 (0.1)	10/60 (16.7)
Cough	1,621/53,819 (3)	99/1,621 (6.1)
Sub total	53,819 (100)	19,217/53,819 (35.7)
**Gastrointestinal**		
Bacterial intestinal infections or intoxications	199/2,706 (7.4)	127/199 (63.8)
Viral enteritis	46/2,706 (1.7)	4/46 (8.7)
GE & colitis	2,412/2,706 (89.1)	1,614/2,412 (66.9)
Appendicitis	21/2,706 (0.8)	2/21 (9.5)
Other	9/2,706 (0.3)	2/9 (22.2)
Sialoadenitis	19/2,706 (0.7)	16/19 (84.2)
Sub total	2,706 (100)	1,765/2,706 (65.2)
**Skin**		
Infective dermatitis	85/4,060 (2.1)	70/85 (82.4)
Dermatophytosis	902/4,060 (22.2)	92/902 (10.2)
Other superficial mycoses	197/4,060 (4.9)	14/197 (7.1)
Candidiasis	101/4,060 (2.5)	23/101 (22.8)
Other	64/4,060 (1.6)	52/64 (81.3)
Scabies & infestations	52/4,060 (1.3)	8/52 (15.4)
Cellulitis & abscesses	841/4,060 (20.7)	618/841 (73.5)
Bacterial skin infections	533/4,060 (13.1)	464/533 (87.1)
Furuncles, caruncles & cysts	947/4,060 (23.3)	780/947 (82.4)
Other local infection of the skin & subcutaneous tissue	338/4,060 (8.3)	290/338 (85.8)
Sub total	4,060 (100)	2,411/4060 (59.4)
**Eye**		
Conjunctivitis	2,097/2,698 (77.7)	330/2,097 (15.7)
Hordeolum & chalazion	319/2,698 (11.8)	256/319 (80.3)
Other inflammation of the eyelid & orbit	268/2,698 (9.9)	98/268 (36.6)
Trachoma	14/2,698 (0.5)	5/14 (35.7)
Sub total	2,698 (100)	689/2,698 (25.5)
**Ear**		

For peer review only

Otitis externa	464/753 (61.6)	369/464 (79.5)
Otitis media	243/753 (32.3)	197/243 (81.1)
Mastoiditis	16/753 (2.1)	9/16 (56.3)
Perforation of tympanic membrane & other disorders	30/753 (4)	25/30 (83.3)
Sub total	753 (100)	600/753 (79.7)
**Urogenital**		
Acute tubulo-interstitial nephritis	36/1,871 (1.9)	32/36 (88.9)
Other	17/1,871 (0.9)	12/17 (70.6)
Cystitis, UTI, dysuria, urethritis & urethral syndrome	1,370/1,871 (73.2)	1,291/1,370 (94.2)
Other disorders of male genital organs	32/1,871 (1.7)	20/32 (62.5)
Other inflammatory disorders of female pelvic organs	148/1,871 (7.9)	115/148 (77.7)
Other inflammatory disorders of the vagina & vulva	268/1,871 (14.3)	149/268 (55.6)
Sub total	1,871 (100)	1,619/1,871 (86.5)
**Other**		
Bacterial	85/3,208 (2.7)	28/85 (32.9)
Unknown aetiology	33/3,208 (1)	14/33 (42.4)
Viral	728/3,208 (22.7)	153/728 (21)
Fungal	36/3,208 (1.1)	2/36 (5.6)
Protozoal	10/3,208 (0.3)	0/10 (0)
Parasitic	1,880/3,208 (58.6)	99/1,880 (5.3)
Nausea & vomiting	268/3,208 (8.4)	30/268 (11.2)
Fever of unknown or other origin	168/3,208 (5.2)	10/168 (6)
Sub total	3,208 (100)	336/3,208 (10.5)

Table S4: the number of presentations per diagnosis and system and whether antibiotics were prescribed for that illness episode

For peer review only

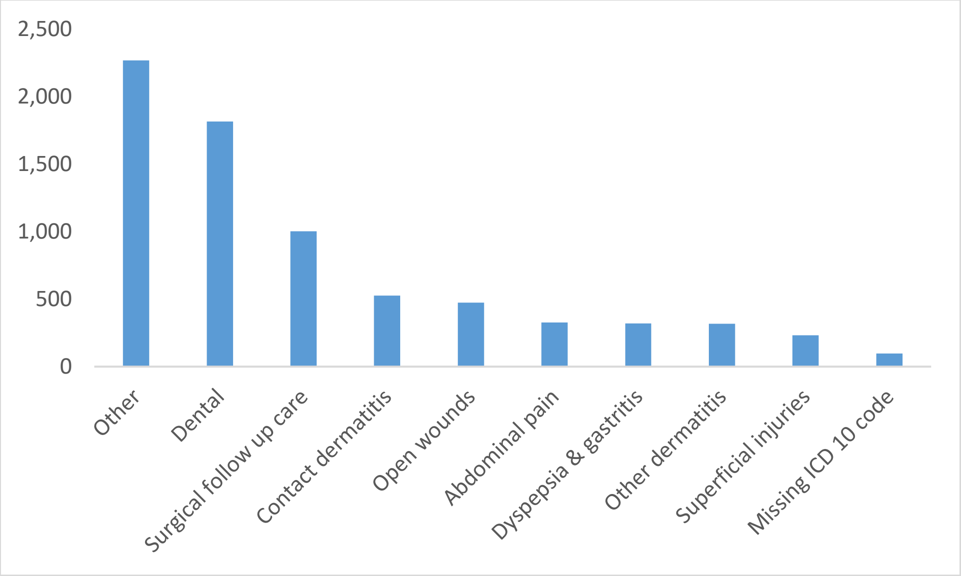

Figure S1: Single diagnoses used for antibiotic prescriptions without a history of fever, temperature or ICD 10 code for infection

For peer review only

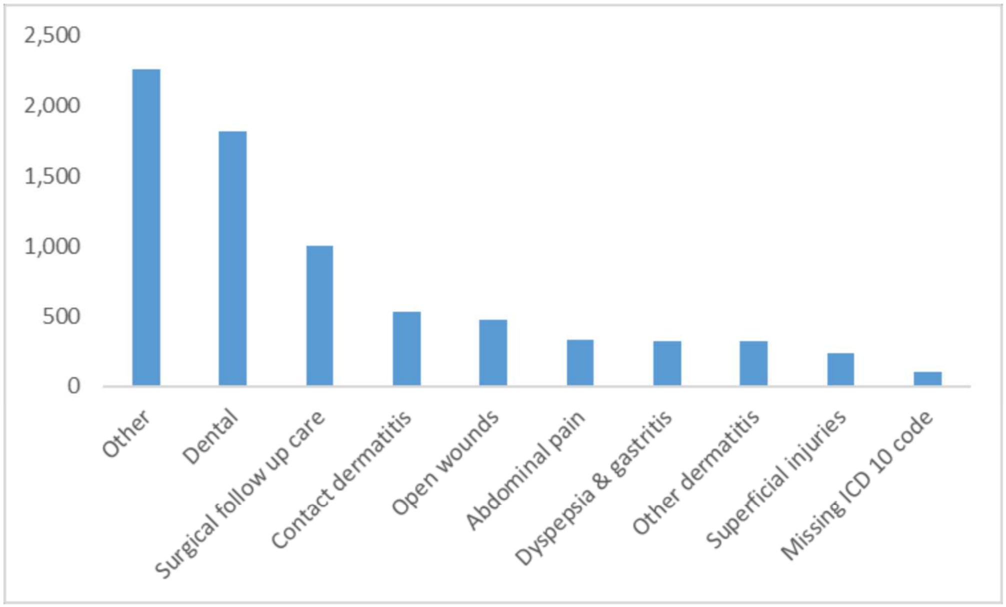

Figure S1: Single diagnoses used for antibiotic prescriptions without a history of fever, temperature or ICD 10 code for infection

127x76mm (300 x 300 DPI)

For peer review only - http://bmjopen.bmj.com/site/about/guidelines.xhtml

BMJ Open

A Retrospective Review of the Management of Acute Infections and the Indications for Antibiotic Prescription in Primary Care in Northern Thailand

Journal:	*BMJ Open*
Manuscript ID	bmjopen-2018-022250.R1
Article Type:	Research
Date Submitted by the Author:	23-Apr-2018
Complete List of Authors:	Greer, Rachel; Mahidol University, Mahidol-Oxford Tropical Medicine Research Unit, Faculty of Tropical Medicine; University of Oxford, Centre for Tropical Medicine and Global Health, Nuffield Department of Medicine,<br>Intralawan, Daranee; Chiangrai Regional Hospital, Social and Preventive Medicine Department<br>Mukaka, Mavuto; Mahidol University, Mahidol-Oxford Tropical Medicine Research Unit, Faculty of Tropical Medicine; University of Oxford, Centre for Tropical Medicine and Global Health, Nuffield Department of Medicine,<br>Wannapinij , Prapass; Mahidol University, Mahidol-Oxford Tropical Medicine Research Unit, Faculty of Tropical Medicine<br>Day, Nicholas; Mahidol University, Mahidol-Oxford Tropical Medicine Research Unit, Faculty of Tropical Medicine; University of Oxford, Centre for Tropical Medicine and Global Health, Nuffield Department of Medicine,<br>Nedsuwan, Supalert; Chiangrai Regional Hospital, Social and Preventive Medicine Department<br>Lubell, Yoel ; Mahidol University, Mahidol-Oxford Tropical Medicine Research Unit, Faculty of Tropical Medicine; University of Oxford, Centre for Tropical Medicine and Global Health, Nuffield Department of Medicine,
**Primary Subject Heading**:	Infectious diseases
Secondary Subject Heading:	General practice / Family practice
Keywords:	antibiotic use, PRIMARY CARE, Thailand, fever, Respiratory infections < THORACIC MEDICINE

SCHOLARONE™
Manuscripts

A RETROSPECTIVE REVIEW OF THE MANAGEMENT OF ACUTE INFECTIONS AND THE INDICATIONS FOR ANTIBIOTIC PRESCRIPTION IN PRIMARY CARE IN NORTHERN THAILAND

Rachel C. Greer[1, 2], Daranee Intralawan[3], Mavuto Mukaka[1, 2], Prapass Wannapinij[1], Nicholas P.J. Day[1, 2], Supalert Nedsuwan[3], Yoel Lubell[1, 2]

[1] Mahidol-Oxford Tropical Medicine Research Unit, Faculty of Tropical Medicine, Mahidol University, Bangkok, Thailand
[2] Centre for Tropical Medicine and Global Health, Nuffield Department of Medicine, University of Oxford, Oxford, United Kingdom
[3] Social and Preventive Medicine Department, Chiangrai Regional Hospital, Chiangrai, Thailand

Corresponding author

Rachel Claire Greer. Address: Mahidol-Oxford Tropical Medicine Research Unit, Faculty of Tropical Medicine, Mahidol University, 3rd Floor, 60th Anniversary Chalermprakiat Building, 420/6 Rajvithi Road, Ratchathewi District, Bangkok 10400, Thailand. Email: rachel@tropmedres.ac Phone: +66 52 029842

Word count: 4002

For peer review only

ABSTRACT

Introduction Antibiotic use in low and middle income countries continues to rise despite the knowledge that antibiotic overuse can lead to antimicrobial resistance. There is a paucity of detailed data on the use of antibiotics in primary care in low resource settings.

Objective Describe the presentation of acute infections and the indications for antibiotic prescription.

Design A two year retrospective review of routinely collected data.

Setting All 32 primary care units in one district in northern Thailand.

Participants Patients attending primary care with a history of fever, documented temperature, ICD 10 code for infection or prescribed a systemic antibiotic. Patients attending after the initiation of a study on C-reactive protein testing in four centres were excluded.

Outcome measures The proportion of patients prescribed an antibiotic and the frequency of clinical presentations.

Results 762,868 patients attended the health centers, of whom 103,196 met the inclusion criteria, 5,966 were excluded resulting in 97,230 attendances consisting of 83,661 illness episodes.

46.9% (39,242) of patients were prescribed an antibiotic during their illness. Indications for antibiotic prescription in the multivariable logistic regression analysis included male sex (aOR 1.21 [CI 1.16-1.28], p <0.001), adults (aOR 1.77 [CI 1.57-2, p <0.001]) and a temperature >37.5°C (aOR 1.24 [CI 1.03-1.48, p 0.020]). 77.9% of the presentations were for respiratory related problems, of which 98.6% were upper respiratory tract infections. The leading infection diagnoses were common cold (50%), acute pharyngitis (18.9%) and acute tonsillitis (5%), which were prescribed antibiotics in 10.5%, 88.7% and 87.1% of cases respectively. Amoxicillin was the most commonly prescribed antibiotic.

Conclusions Nearly half of the patients received an antibiotic, the majority of whom had a respiratory infection. The results can be used to plan interventions to improve the rational use of antibiotics. Further studies in private facilities, pharmacies and dental clinics are required.

Word count: 299

Keywords: antibiotic use, primary care, Thailand, fever, respiratory infections

ARTICLE SUMMARY

Strengths and limitations of this study

- Over 80,000 illness episodes reviewed from all primary care units in a district, over a two year time period
- Wide range of infections included rather than focusing on one specific infection
- Use of routine electronic data (no Hawthorne Effect), making this work reproducible
- Only included public healthcare facilities
- Reliant on the correct coding and clinical diagnoses of illnesses

For peer review only

BACKGROUND

The proportion of global deaths attributable to communicable diseases has greatly reduced in recent years. Despite these improvements, 10.6% of deaths worldwide in 2015 were thought to be caused by lower respiratory tract infections (LRTIs), diarrhoea, and tuberculosis (TB).[1] In under five year olds, 51.8% of deaths worldwide were due to infectious causes in 2013, with pneumonia causing 14.8% of the deaths overall.[2] In Thailand in 2010, respiratory infections were the leading cause of hospitalisations and deaths in children under the age of five.[3] Prompt access to appropriate antibiotics is vital to prevent many of these unnecessary deaths.[4] But while inappropriate or no treatment remains a clear cause for concern, the global antibiotic consumption rate increased by 39% between 2000 and 2015, fueled by low and middle income countries (LMIC),[5 6] with the majority of antibiotics being consumed in the community.[7]

Overuse and misuse of antibiotics have been linked to the development of antimicrobial resistance (AMR).[7-9] Antibiotics prescribed to individuals in primary care have been associated with bacterial resistance in that individual for up to 12 months, and longer and more frequent antibiotic courses are more likely to cause resistance.[10] The World Health Organization has described AMR in Southeast Asia as a 'burgeoning and often neglected' issue, stating that a 'post-antibiotic era' may become reality, resulting in common infections and minor injuries being untreatable.[11] In Thailand in 2010, there were an estimated 19,122 deaths attributable to multidrug resistant hospital-acquired infections.[12] Thailand has been making sustained efforts to reduce inappropriate antibiotic use; its Antibiotic Smart Use program started in 2007 and targets three conditions which are unlikely to require antibiotic treatment but for which they are commonly prescribed: upper respiratory tract infections (URTIs), acute diarrhoea and simple wounds.[13] Prescribing targets have been incorporated into the public health system's pay for performance criteria. In August 2016 the Thai government endorsed a national strategic plan for AMR which aims to optimize antimicrobial drug use and reduce the mean consumption of antimicrobials in humans by 20% by 2021.[14]

To appreciate the scale of the problem and to target future interventions, a greater understanding of the acute infections presenting to primary care and the conditions for which antibiotics are used in LMICs is required. Such data, however, are limited,[15] with most studies deriving their estimates from small samples of health providers and over a limited timeframe, therefore neglecting possible seasonal and spatial variation and other secular trends. In this paper we describe the indications for antibiotic prescription in 32 primary care units (PCUs) across a district in northern Thailand over a two year period.

Study sites

Thailand is an upper-middle income country. In 2016 its gross domestic product (GDP) was 406.8 billion USD. The average life expectancy at birth is 75 years.[16] Chiangrai is the most northern province in Thailand and shares borders with Laos and Myanmar. It has a population of 1,282,544, of whom 241,436 reside in Mueang Chiangrai District.[17 18] Thailand has three seasons, the wet season typically runs from July to October, the cool season from November to February and the hot season from March to June.

Thailand's healthcare system is made up of public and private providers. Universal health coverage was established in 2002 following significant investment in the healthcare system and infrastructure since the 1970s. In rural and poorer areas primary healthcare is predominantly provided by the public healthcare system whereas in urban areas hospitals and private clinics play a larger role.[19]

For peer review only

Antibiotics can be bought directly from pharmacies and local stores as well as being prescribed by healthcare workers. Community antibiotic guidelines exist for some but not all common infections, including assessment criteria (e.g. the Centor criteria for acute tonsillitis), first line antibiotics, their dose and duration. There are prescribing restrictions in place for some broad spectrum antibiotics such as amoxicillin and clavulanic acid (Co-amoxiclav), which cannot be prescribed by nurses working in the public primary care system. More comprehensive hospital based guidelines are available.

In Mueang Chiangrai District family medicine doctors based at the provincial hospital oversee 32 public PCUs which are staffed primarily by two to five nurses and public health officers. On average PCUs look after 5000 patients each.[19] They provide care for acute and chronic conditions as well as providing preventative services such as immunisations, cervical screening and health education. Dental and traditional medicine services are also available. The furthest PCU is two hours' drive through the mountains from the provincial hospital in Chiangrai city (see figure 1). Finger-prick blood glucose tests are the only investigations routinely available on site.

METHOD

A retrospective computerised search of routinely collected data from primary care units in Mueang Chiangrai District between January 2015 and December 2016 was carried out.

Inclusion criteria

Patients were identified with at least one of the following:

- Systemic antibiotic prescription
- International Statistical Classification of Diseases (ICD) 10 code for infection (see supplementary material, table S1)
- Fever as the chief complaint
- Documented temperature >37.5°C at the PCU

We excluded patients attending PCUs used as study sites during or after a recent trial on the use of C-reactive protein (CRP) point of care tests (https://www.clinicaltrials.gov/ct2/show/NCT02758821?term=NCT02758821&rank=1).

Study outcomes

The primary outcome was the overall proportion of illness episodes prescribed an antibiotic. Risk factors for antibiotic use are reported as well as the percentages of patients receiving antibiotics according to their diagnosis, percentages of individual antibiotics used and the frequency and type of acute infection presentations.

Data collection

With the approval of the Chiangrai Provincial and Public Health Office (PHO), a research data manager accessed the PHO's routine medical records database to search for relevant patients and extract the pre-specified variables. Data collected consisted of the PCU attended, patients' number, age, sex, date of visit, chief complaint, temperature, ICD 10 code, and drug prescriptions.

Data cleaning and coding

For peer review only

Each inclusion criteria was classified as being present, absent or that the data were missing. Antibiotics were searched for in the prescription field (free text) and coded as prescribed (yes or no). A predefined antibiotic list (see supplementary material) was generated using the formulary. All medications prescribed in the dataset were reviewed to ensure no antibiotics were omitted due to spelling errors or their absence from our original list. If no prescription was recorded we made the assumption that this was because no medication was given rather than the data being missing.

Our predefined list of ICD 10 codes for infection (see supplementary material, table S1) were searched for in the diagnosis field (free text field, containing ICD 10 codes only) and then coded as present or absent. Our list of ICD 10 codes were reviewed with the other variables to ensure their appropriateness.

We searched for the word 'fever' in Thai in the chief complaint field (free text). In some cases 'no fever' was recorded, or the word 'fever' was present but part of a phrase alluding to a patient more generally, or 'influenza vaccine'. This field was checked manually with the help of two native northern Thai speaking study nurses. History of fever in the chief complaint was then coded as yes or no. Documented temperatures over 37.5°C at the PCU were searched for in the temperature field and then coded as temperature >37.5°C yes or no.

Repeat attendances within one month were classed as one illness episode allowing for the detection of subsequent antibiotics or treatment changes. All other indications such as the chief complaint and temperature were taken from the initial presentation. Children were defined as being under 12 years of age. The ICD 10 codes were grouped into gastrointestinal, respiratory, skin, urogenital, eye, ear and other categories. Each category was further broken down into conditions such as acute sinusitis and acute pharyngitis. The respiratory category was also grouped into upper and lower respiratory tract infections.

Statistical analyses

Descriptive statistics

Categorical variables were summarised using counts and percentages. Non-normally distributed data were described using medians and interquartile ranges and compared using the ranksum test. The proportions of patients prescribed an antibiotic in different demographic groups were summarised and compared using the chi-square test.

Logistic and Poisson regression models

A logistic regression model was used to model the binary outcome of antibiotic prescription (yes or no); both unadjusted and adjusted models were fitted and accounted for clustering of patients attending the same PCU. The odds ratios for the indications of antibiotic prescription were first obtained from univariate logistic regression models and then considered in a multivariable model if they had a p value of <0.05. Indications included sex, age category and documented temperature. The main purpose of this model was to identify risk factors that were independently associated with antibiotic prescription. A temperature of >37.5°C was used rather than the more subjective history of fever. ICD 10 codes were not included because of their strong association with antibiotic prescriptions (e.g. a health worker's diagnosis of acute pharyngitis and its affiliated ICD 10 code was inherently associated with antibiotic prescription, as opposed to a diagnosis of 'common cold'). Furthermore a Poisson regression model of

For peer review only

the monthly number of antibiotic prescriptions over the 24 month period was produced to obtain the Incidence Rate Ratios and 95% confidence intervals.

Time-series analysis

Monthly antibiotic prescriptions were weighted by the number of contributing PCUs per month and modelled over the two year period. When time-series analysis is used for forecasting, it is common to apply it to periods of five years or more, however, our aim was not to forecast into the future but to simply describe the current trends in antibiotic prescription.[20 21] We used a time-series analysis to separate long-term trends from seasonal variations.[22 23] Symmetric Locally Weighted Moving Averages (MA) were used. In this procedure, less weight was applied to time points (in months) furthest away from the present time point. The data was available on a monthly basis, however, a quarterly window was used to identify seasonality as follows: $\hat{X}_t = \frac{1}{9}(X_{t-2} + 2X_{t-1} + 3X_t + 2X_{t+1} + X_{t+2})$

Similarly a 12-month time-series window was used to obtain a trend line that would be sensitive to monthly changes but with reduced noise from seasonal variation:

$$\hat{X}_t = \frac{1}{24}(X_{t-6} + X_{t+6}) + \frac{1}{12}(X_t + X_{t-1} + X_{t+1} + X_{t-2} + X_{t+2} + X_{t-3} + X_{t+3} + X_{t-4} + X_{t+4} + X_{t-5} + X_{t+5})$$

Where $\hat{X}_t$ is the time-series modelled monthly prevalence of antibiotic prescription. Statistical significance was declared at alpha=0.05. Data analyses were performed with STATA version 14 (College Station, Texas, USA).

Ethical approval

Ethical approval was obtained from Chiangrai's Provincial and Public Health Office IRB (number 56/2560). Exemption was given by the Oxford Tropical Research Ethics Committee (OxTREC).

Patient and public involvement

Patients were not involved in the design of the study. Due to the study's retrospective nature patients were not involved in the recruitment processes. Study results will be disseminated through community presentations as well as educational updates for the healthcare workers and community volunteers.

RESULTS

762,868 patients attended the PCUs between the 1st of January 2015 and the 31st of December 2016. The majority of patients' attendances included a chronic disease review or screening, the most common being screening for diseases such as diabetes, hypertension, mental health and dental disorders (145,410), essential hypertension reviews (98,822) and routine child health examinations (75,701).

For peer review only - http://bmjopen.bmj.com/site/about/guidelines.xhtml

The appropriateness of the ICD 10 codes for infection used in our inclusion criteria were reviewed alongside the other variables. For example, we found that patients with TB, HIV and Hepatitis B were only attending for regular medications rather than for acute illnesses so they were removed from the ICD 10 inclusion list. Mass head lice treatment at schools is carried out by the PCUs so these codes were also removed. The ICD 10 code 'K05' (dental) was also removed because it transpired that these patients are seen by dentists or dental nurses at the PCUs rather than by the regular PCU staff. All ICD 10 codes for myositis were removed from the inclusion criteria apart from M60.0 (infective myositis) because the other codes were being used for muscle pain or myalgia (see supplementary material, table S1).

In total 103,196 attendances met our inclusion criteria; 5,966 were then excluded because the PCUs they attended were involved in the CRP study before or during their attendance, resulting in 97,230 attendances (12.7%) meeting our inclusion and exclusion criteria. 13,569 repeat attendances within one month were classed as a single illness episode, leaving 83,661 illness episodes.

Patient characteristics

The median age was 24 years old with an interquartile range of 6 to 51 years old. Two patients had no age recorded. 54.7% of the patients were female (45,779) compared to 45.3% males (37,882) ($p<0.001$).

The proportion of patients meeting each inclusion criteria is shown in figure 2 and supplementary material table S2. 29,246 (35.3%) patients presented with a history of fever, while 10,508 (13.7%) had a temperature of more than 37.5°C at presentation. 8,871 (11.6%) patients had both a history of fever and a temperature.

Antibiotics

Medications were prescribed for 81,691 (97.7%) illness episodes. 37,011 (44.2%) patients were prescribed an antibiotic during their first visit, and this increased to 39,242 (46.9%) throughout their illness episodes.

Antibiotics were prescribed to:

- 49.2% of men compared to 45% of women ($p<0.001$)
- 39% of children compared to 51.8% of adults ($p <0.001$)
- 40.1% of those with a history of fever
- 47.6% with a temperature >37.5°C
- 38.8% with an ICD 10 code for infection

The proportion of patients within each age group prescribed an antibiotic varied, with the lowest rates in young children (0-4 year olds, 33.8%), peaking in adults (12-39 year olds, 55.9%) and then diminishing in the elderly (aged 65 years and older, 41%, see supplementary material, table S3).

The odds ratios for the univariate and multivariable logistic regression analyses are shown in table 1. All variables entered into the univariate model were significant so were added to the multivariable analysis. Indications for antibiotic prescription in the adjusted multivariable logistic regression analysis were male sex (aOR 1.21 [CI 1.16-1.28], $p <0.001$), patients aged 12 years of age or older (compared to those less than 12 years old) (aOR 1.77 [CI 1.57-2, $p <0.001$]) and having a temperature of more than 37.5°C (aOR 1.24 [CI 1.03-1.48, p 0.02]).

For peer review only

Variable	OR (confidence interval)	p value	aOR (confidence interval)	p value
**Univariate analysis**			**Multivariable analysis**	
**Male sex**	1.18 (1.12-1.25)	<0.001	1.21 (1.16-1.28)	<0.001
**Aged ≥ 12 years old**	1.68 (1.48-1.90)	<0.001	1.77 (1.57-2)	<0.001
**Temperature >37.5°C**	1.05 (0.85-1.30)	0.197	1.24 (1.03-1.48)	0.020

Table 1: Univariate and multivariable logistic regression analyses accounting for clustering of patients attending the same PCU, showing all included variables and their association with antibiotic prescription

Figure 3 is a time series plot for the monthly prevalence of antibiotic prescriptions. Overall there was no significant trend; incidence rate ratio (IRR) =0.99, 95% CI (0.990, 1.007), p=0.796, although there is a suggestion of a downward trend beginning in the final 6 months The monthly prevalence of antibiotic prescriptions was at least 39% throughout the two year period. Patients attending in the wet season (July-October) were more likely to receive antibiotics (47.4%) than those attending in the hot and cold seasons (46.6%) p value 0.029. Overall prescription rates varied greatly between the PCUs from 8 to 71.6%, with prescribing consistently higher in adults than in children.

The majority of patients prescribed an antibiotic received amoxicillin (56.7%) or dicloxacillin (25.1%). Other antibiotics prescribed include norfloxacin (8.9%), co-trimoxazole (4.2%), penicillin V (1.2%), roxithromycin (1.2%), metronidazole (1.2%), erythromycin (0.7%), cephalexin (0.4%) and tetracycline (0.2%).

Presentations and antibiotic prescriptions

The number of acute presentations with ICD 10 codes for infection related to a single system are shown in figure 4. 77.9% of these presentations were for respiratory related problems. 98.6% of these were diagnosed with an URTI, 1.1% with an acute LRTI and 0.3% with a chronic LRTI, of these 36.1%, 81.8% and 53.5% were prescribed antibiotics respectively. The most common single infection diagnoses were common cold (34,549, 50%), acute pharyngitis (13,080, 18.9%) and acute tonsillitis (3,459, 5%), antibiotics were prescribed to 10.5%, 88.7% and 87.1% of the cases respectively (see table 2).

Diagnosis	Number of presentations n/N (%)	Episode antibiotics prescribed n/N (%)	Commonest antibiotic prescribed (%)
**Common cold**	34,549/69,115 (50)	3,643/34,549 (10.5)	Amoxicillin (71.7)
**Acute pharyngitis**	13,080/69,115 (18.9)	11,607/13,080 (88.7)	Amoxicillin (91.5)
**Acute tonsillitis**	3,459/69,115 (5)	3,014/3,459 (87.1)	Amoxicillin (93.4)
**Gastroenteritis & colitis unspecified**	2,412/69,115 (3.5)	1,614/2,412 (66.9)	Norfloxacin (68.8)
**Conjunctivitis**	2,097/69,115 (3.0)	330/2,097 (15.7)	Amoxicillin (56.4)
**Other helminthiases**	1,231/69,115 (1.8)	65/1,231 (5.3)	Amoxicillin (41.5)
**Cystitis**	1,230/69,115 (1.8)	1,165/1,230 (94.7)	Norfloxacin (75.9)

For peer review only

Table 2: Common diagnoses in patients with one single ICD 10 code for infection, whether antibiotics were prescribed and which antibiotic was mostly commonly used

Supplementary table S4 shows the number of individual infection diagnoses by systems and the rates of antibiotic prescriptions. Antibiotics were prescribed to 59.4% of skin infections, 81.1% of otitis media, 79.5% of otitis externa, 94.7% of cystitis, 80.3% of hordeolum (styes) and chalazions as well as 15.7% of conjunctivitis cases. Of the total antibiotics prescribed almost a third (29.6%) were given to those with acute pharyngitis, followed by common cold (9.3%), acute tonsillitis (7.7%), gastroenteritis and colitis (4.1%) and cystitis (3%) as the single infection diagnoses.

13.8% of patients (11,547) were prescribed antibiotics without a temperature, history of fever or ICD 10 code for infection. Of those who had a single ICD 10 code recorded 1,815 (24.6%) of these antibiotics were for dental reasons, 1,002 (13.6%) for surgical follow up care, 526 (7.1%) for contact dermatitis and 473 (6.4%) for open wounds (see supplementary material, figure S1). These patients were more likely to be male (54.3%, p value <0.001) and older (median age of 41 compared to 24 years old) than the main patient group.

DISCUSSION

To the best of our knowledge this is the largest review of acute illness presentations and community antibiotic prescribing in a LMIC. Over a two year period there were more than 97,000 attendances to 32 PCUs for acute infections and nearly half these patients received an antibiotic, with no significant change in prescribing levels over the two year study period. Studies of this magnitude are required to increase our knowledge of the scale of antibiotic prescribing as well as the common conditions they are used for.[24 25] Thailand's 2016 national strategic plan on AMR also highlighted the importance of monitoring and reporting antimicrobial consumption.[26]

Comparing overall antibiotic prescribing rates with other studies is challenging because of varying definitions of acute illnesses and the different patient populations. However the antibiotic prescribing rate in our study is more than double the prescribing in a Malaysian study but similar to studies in India and Laos.[27-29] A third of our patients had a history of fever, which is similar to a point prevalence study in India where fever was the most common symptom.[30] Almost 80% of the ICD 10 codes for infection were related to respiratory infections, which is consistent with respiratory infections being the leading cause of hospitalisations and deaths in Thai under five year olds[3] but is higher than the proportion of respiratory presentations in other South and Southeast Asian countries.[30 31] Antibiotic prescribing in Thailand for tonsillitis and pharyngitis remains high despite Group A beta-haemolytic Streptococci being isolated in only 3.8-7.9% of those with URTI.[32 33]

In the first phase of Thailand's Antibiotic Smart Use program overall antibiotic use in PCUs was reduced by between 39% and 46%. Prescriptions for the three target conditions (URTI, acute diarrhoea and simple wounds) reduced from 54.5% to 25.4%.[34] Despite the lower prescribing levels of 10.5% for common colds in our review, there were still 3,643 antibiotic prescriptions for this condition, alongside 88.7% of those with acute pharyngitis, 87.1% with acute tonsillitis and 66.9% with gastroenteritis and colitis receiving antibiotics; this is likely to represent the overuse of antibiotics. Open wounds and superficial injuries were common diagnoses in those prescribed an antibiotic without a history of fever,

For peer review only

temperature, or ICD 10 code for infection. The results reveal the ongoing high levels of prescribing for these conditions and present an opportunity to further reduce antibiotic use. Since late 2016 an antibiotic prescribing target of less than 20% for these three conditions has been incorporated into Thailand's rational drug use service plan as well as the pay for performance health criteria, and financial incentives are given to the PCUs achieving this target. A review of the long term effectiveness of this policy including any impact on patient safety is required.

Our study also identifies high levels of prescribing for skin infections, otitis media, otitis externa, cystitis, hordeolum (styes) and chalazions. A lack of available topical antibiotics may account for the high prescribing for skin infections as well as for otitis externa. However, despite antibacterial eye drops being available, 15.7% of conjunctivitis cases were still prescribed a systemic antibiotic. Urine dipstick tests are not available on site to assess patients with cystitis or suspected urinary tract infections. Introduction of these simple tests may help to rationalize prescribing for these conditions in a setting where urine cultures are not readily available or achievable.

While we did not set out to review dental prescribing, this area accounted for 25% of the antibiotics prescribed to those without a history of fever, temperature or ICD 10 code for infection, which warrants further investigation.

Some of the variation in antibiotic prescribing rates between PCUs may be accounted for by the degree of staff training. Two out of the three highest prescribing PCUs are staffed only by public health officers. The study findings are being used to guide educational updates and training for the PCU staff, with priority being given to those PCUs without nurses and with high prescription rates for conditions unlikely to require antibiotics.

A wide range of antibiotics are prescribed in the PCUs. Restrictions are in place for some broad-spectrum antibiotics such as amoxicillin and clavulanic acid (Co-amoxiclav) which cannot be prescribed. One area of concern is that less than 1% of the antibiotics being prescribed have activity against scrub typhus, which is the leading cause of hospital admission with acute undifferentiated fever in this region.[35]

Strengths and limitations

The main strength of this study is the large number of illness episodes included. The two year time period should allow for seasonal variations and disease epidemics. We reviewed prescribing in all of the PCUs in Mueang Chiangrai District which covers a large geographical area and has a range of rural and urban facilities, making the results generalizable to the region more broadly. Many studies have focused on prescribing for specific conditions such as URTIs but our study covers a wide range of infections that present in the community. Having research staff on site has been shown to influence healthcare workers' prescribing habits (the Hawthorne Effect), but due to the retrospective nature of the study this was not a source of bias. The use of routinely collected data means that this methodology could be repeated in other districts and provinces in Thailand, although a lot of the data are entered as free text which presents challenges for analysis. Searching for patients with a history of fever, for instance, was problematic because the Thai word 'ไข้' or fever is also part of the Thai words for patient, influenza, anti-pyrexials, etc.

Limitations of this study are that we only included public PCUs and have no data on antibiotic use by private clinics, pharmacies or family medicine doctors based at the provincial hospital, which requires further study. The PCU data is taken from routine electronic records and in some instances there were tranches of missing data (five PCUs had no recorded data for several months). Verifying the quality of some data is also challenging; coding of clinical diagnoses for instance using ICD 10 could be inconsistent between healthcare workers and in primary care the majority of infections are diagnosed clinically without any laboratory tests. However we used data from a subsample of patients enrolled in a clinical trial in four PCUs and compared them to their respective routine medical records. While minor discrepancies were found in their precise age and temperature the diagnoses and antibiotic prescribing data were consistent. Our decision to class all attendances within a one month period as a single illness episode means that we may have incorrectly classed some new illnesses as a repeat attendance but did allow us to review antibiotic prescribing over the course of the illness. The time series analysis was carried out using data from a two year time period, more definitive conclusions and trends may have become apparent if further time points and data were available.

CONCLUSIONS

This study provides much needed insight into the use of antibiotics in primary care in northern Thailand, allowing targeting of interventions to improve the rational use of antibiotics. Nearly half of all patients attending with an acute illness received an antibiotic. The majority of presentations were for respiratory infections. Further education and resources are required to support clinicians in the targeting of antibiotics. This could include the introduction of clinical algorithms and point of care tests such as CRP and urine dipsticks. Antibiotic guidelines are required for common conditions seen in primary care outside of the current Antibiotic Smart Use policy. Further studies including qualitative work are required to appreciate the use of antibiotics in other settings such as private facilities, pharmacies and dental clinics.

Funding statement

This research received no specific grant from any funding agency in the public, commercial or not-for-profit sectors. This study was part of the Wellcome Trust Major Overseas Programme in SE Asia (grant number 106698/Z/14/Z).

Competing interests

None declared

Author contribution statement

All authors were involved in the design of the study. PW collected the data. RCG carried out the analysis with support from YL. MM provided statistical support. RCG, YL, DI, SN interpreted the data. RCG wrote

For peer review only

the first draft of the paper. YL, NPJD and MM reviewed subsequent drafts. All authors approved the final draft for publication.

Acknowledgements

We would like to thank Wasuphal Kanjana for his help with data collection, Nipaphan Kanthawang and Pratakpong Wongkiti for their help with data cleaning and translation of the Thai abstract and Areerat Thaiprakhong for her assistance with figure 1.

Data sharing statement

We are unable to share additional unpublished data which falls under the jurisdiction of the Chiang Rai PHO.

Ethical approval

Ethical approval was obtained from Chiangrai's Provincial and Public Health Office IRB (number 56/2560). Exemption was given by the Oxford Tropical Research Ethics Committee (OxTREC). Individual informed consent was not required.

For peer review only

REFERENCES

1. The World Health Organization. The top 10 causes of death 2017 [Available from: http://www.who.int/mediacentre/factsheets/fs310/en/(Accessed January 2018)
2. Liu L, Oza S, Hogan D, et al. Global, regional, and national causes of child mortality in 2000-13, with projections to inform post-2015 priorities: an updated systematic analysis. *Lancet* 2015;385(9966):430-40. doi: 10.1016/S0140-6736(14)61698-6 [published Online First: 2014/10/05]
3. Teeratakulpisarn J, Uppala R, Thepsuthammarat K, et al. Burden of Acute Lower Respiratory Infection in Children in Thailand in 2010: Have We Achieved the National Target in Under-five Morbidity and Mortality? *Journal of the Medical Association of Thailand = Chotmaihet thangphaet* 2012;95(7):S87-96.
4. Laxminarayan R, Matsoso P, Pant S, et al. Access to effective antimicrobials: a worldwide challenge. *Lancet* doi: 10.1016/S0140-6736(15)00474-2
5. Klein EY, Van Boeckel TP, Martinez EM, et al. Global increase and geographic convergence in antibiotic consumption between 2000 and 2015. *Proceedings of the National Academy of Sciences* 2018
6. Van Boeckel TP, Gandra S, Ashok A, et al. Global antibiotic consumption 2000 to 2010: an analysis of national pharmaceutical sales data. *Lancet Infect Dis*;14(8):742-50. doi: 10.1016/S1473-3099(14)70780-7
7. The European Centre for Disease Prevention and Control (ECDC). Surveillance of antimicrobial consumption in Europe 2012. 2014 [Available from: https://ecdc.europa.eu/sites/portal/files/media/en/publications/Publications/antimicrobial-consumption-europe-esac-net-2012.pdf (Accessed August 2017)
8. Laxminarayan R, Duse A, Wattal C, et al. Antibiotic resistance - the need for global solutions. *Lancet Infect Dis* 2013;13(12):1057-98. doi: 10.1016/S1473-3099(13)70318-9
9. Gelband H, Miller-Petrie M, Pant S, et al. The State of the World's Antibiotics: Center for Disease Dynamocs, Ecomomics & Policy (CDDEP). 2015 [Available from: https://www.cddep.org/publications/state_worlds_antibiotics_2015/ (Accessed October 2017)
10. Costelloe C, Metcalfe C, Lovering A, et al. Effect of antibiotic prescribing in primary care on antimicrobial resistance in individual patients: systematic review and meta-analysis. *BMJ* 2010;340 doi: 10.1136/bmj.c2096
11. The World Health Organization. Antimicrobial Resistance. Global Report on Surveillance 2014 [Available from: http://apps.who.int/iris/bitstream/10665/112642/1/9789241564748_eng.pdf?ua=1 (Accessed March 2017)
12. Lim C, Takahashi E, Hongsuwan M, et al. Epidemiology and burden of multidrug-resistant bacterial infection in a developing country. *eLife* 2016;5:e18082. doi: 10.7554/eLife.18082
13. Sumpradit N, Chongtrakul P, Anuwong K, et al. Antibiotics Smart Use: a workable model for promoting the rational use of medicines in Thailand. *Bulletin of the World Health Organization* 2012;90(12):905-13. doi: 10.2471/BLT.12.105445
14. Tangcharoensathien V, Sattayawutthipong W, Kanjanapimai S, et al. Antimicrobial resistance: from global agenda to national strategic plan, Thailand. *Bulletin of the World Health Organization* 2017;95(8):599-603. doi: 10.2471/BLT.16.179648
15. Zellweger RM, Carrique-Mas J, Limmathurotsakul D, et al. A current perspective on antimicrobial resistance in Southeast Asia. *J Antimicrob Chemother* 2017;72(11):2963-72. doi: 10.1093/jac/dkx260
16. World bank group. Thailand data 2017 [Available from: https://data.worldbank.org/country/thailand (Accessed October 2017)

For peer review only

17. National Statistical Office. Chiang Rai provincial statistical report 2017 [Available from: http://chiangrai.nso.go.th/images/data/%2060.compressed.pdf (Accessed April 2018)
18. Department of Provincial Administration. Official Statistics Registration Systems, 2016. [Available from: http://stat.dopa.go.th/stat/statnew/upstat_age.php] (Accessed November 2017)
19. Asia Pacific Observatory on Health Systems and Policies. The Kingdom of Thailand Health System Review. *Health Sys in Transit* 2015;5(5)
20. Albu JB, Sohler N, Li R, et al. An Interrupted Time Series Analysis to Determine the Effect of an Electronic Health Record–Based Intervention on Appropriate Screening for Type 2 Diabetes in Urban Primary Care Clinics in New York City. *Diabetes Care* 2017;40(8):1058.
21. Girgis A, Kelly B, Boyes A, et al. The PACT study protocol: a time series study investigating the impact, acceptability and cost of an integrated model for psychosocial screening, care and treatment of patients with urological and head and neck cancers. *BMJ Open* 2014;4(1)
22. Moineddin R, Upshur RE, Crighton E, et al. Autoregression as a means of assessing the strength of seasonality in a time series. *Popul Health Metr* 2003;1(1):10. doi: 10.1186/1478-7954-1-10 [published Online First: 2003/12/17]
23. Wall EC, Everett DB, Mukaka M, et al. Bacterial Meningitis in Malawian Adults, Adolescents, and Children During the Era of Antiretroviral Scale-up and Haemophilus influenzae Type b Vaccination, 2000–2012. *Clin Infect Dis* 2014;58(10):e137-e45. doi: 10.1093/cid/ciu057
24. O'Brien K, Bellis TW, Kelson M, et al. Clinical predictors of antibiotic prescribing for acutely ill children in primary care: an observational study. *Br J Gen Pract* 2015;65(638):e585.
25. Petersen I, Hayward AC. Antibacterial prescribing in primary care. *J Antimicrob Chemother* 2007;60(suppl_1):i43-i47. doi: 10.1093/jac/dkm156
26. Sumpradit N, Wongkongkathep S, Poonpolsup S, et al. New chapter in tackling antimicrobial resistance in Thailand. *BMJ* 2017;358
27. Keohavong B, Syhakhang L, Sengaloundeth S, et al. Rational use of drugs: prescribing and dispensing practices at public health facilities in Lao PDR. *Pharmacoepidemiology Drug Saf* 2006;15(5):344-47. doi: 10.1002/pds.1169
28. Ab Rahman N, Teng CL, Sivasampu S. Antibiotic prescribing in public and private practice: a cross-sectional study in primary care clinics in Malaysia. *BMC Infect Dis* 2016;16 doi: 10.1186/s12879-016-1530-2
29. Kotwani A, Holloway K. Trends in antibiotic use among outpatients in New Delhi, India. *BMC Infect Dis* 2011;11:99-99. doi: 10.1186/1471-2334-11-99
30. Salvi S, Apte K, Madas S, et al. Symptoms and medical conditions in 204912 patients visiting primary health-care practitioners in India: a 1-day point prevalence study (the POSEIDON study). *Lancet Glob Health* 2015;3(12):e776-e84. doi: 10.1016/S2214-109X(15)00152-7
31. Mimi O, Tong SF, Nordin S, et al. A Comparison of Morbidity Patterns in Public and Private Primary Care Clinics in Malaysia. *Malaysian Family Physician : the Official Journal of the Academy of Family Physicians of Malaysia* 2011;6(1):19-25.
32. Boonyasiri A, Thamlikitkul V. Effectiveness of multifaceted interventions on rational use of antibiotics for patients with upper respiratory tract infections and acute diarrhea. *Journal of the Medical Association of Thailand* 2014;97(3 SUPPL. 3):S13-S19.
33. Treebupachatsakul P, Tiengrim S, Thamlikitkul V. Upper respiratory tract infection in Thai adults: prevalence and prediction of bacterial causes, and effectiveness of using clinical practice guidelines. *Journal of the Medical Association of Thailand = Chotmaihet thangphaet* 2006;89(8):1178-86.
34. The World Health Organization. The Pursuit of Responsible Use of Medicine: Sharing and Learning from Country Experiences. 2012[Available

For peer review only

from:http://www.who.int/medicines/publications/responsible_use/en/] (Accessed December 2017)
35. Wangrangsimakul T, Althaus T, Mukaka M, et al. Causes of acute undifferentiated fever and the utility of biomarkers in Chiangrai, northern Thailand. Submitted

Figure 1: Chiangrai and the 32 PCUs

Figure 2: A Venn diagram to show the inclusion criteria

Figure 3: Trend and seasonality of antibiotic prescriptions overlaid by mean antibiotic prescription rates per PCU

Figure 4: Number of acute presentations by single systems and whether antibiotics were prescribed

For peer review only

For peer review only

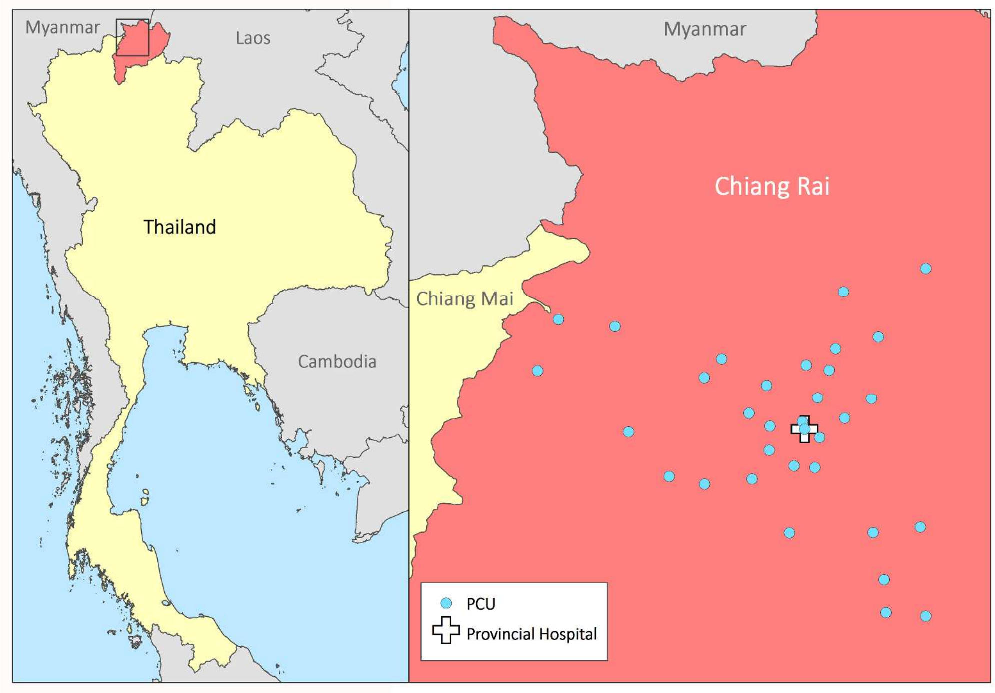

Figure 1: Chiangrai and the 32 PCUs

354x246mm (300 x 300 DPI)

For peer review only

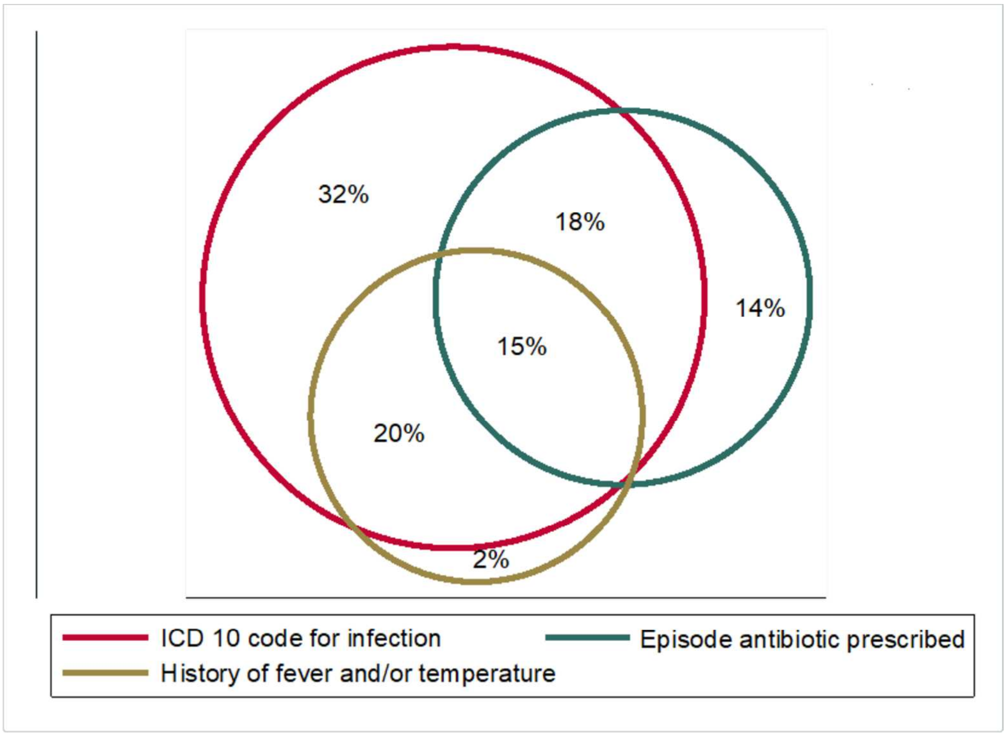

Figure 2: A Venn diagram to show the inclusion criteria

150x109mm (300 x 300 DPI)

For peer review only - http://bmjopen.bmj.com/site/about/guidelines.xhtml

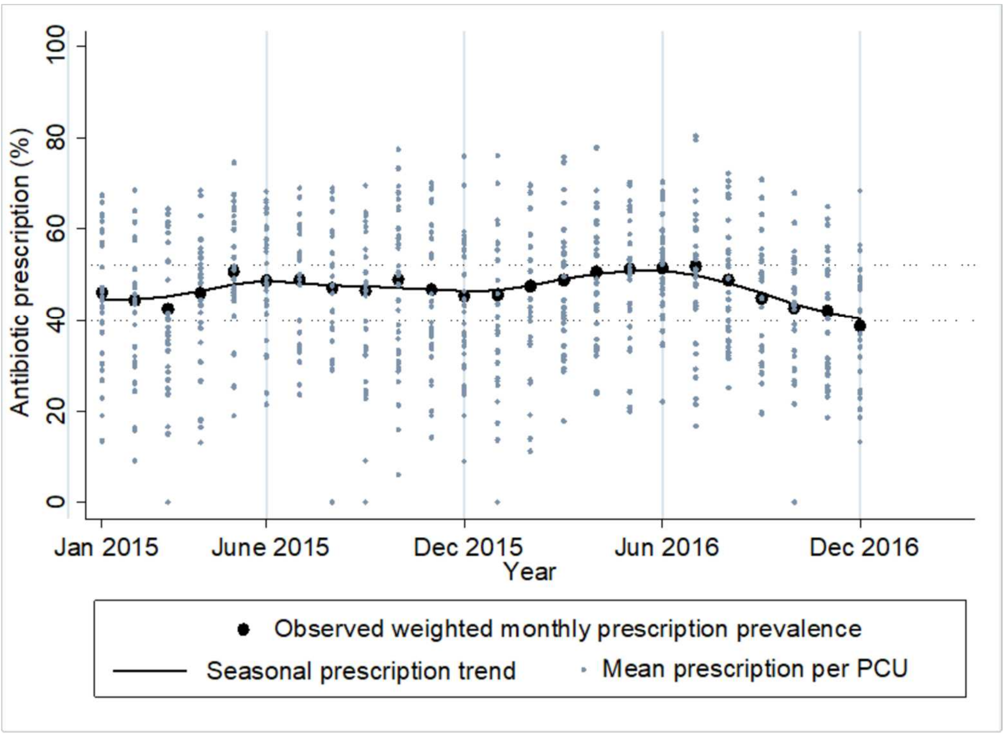

Figure 3: Trend and seasonality of antibiotic prescriptions overlaid by mean antibiotic prescription rates per PCU

127x92mm (300 x 300 DPI)

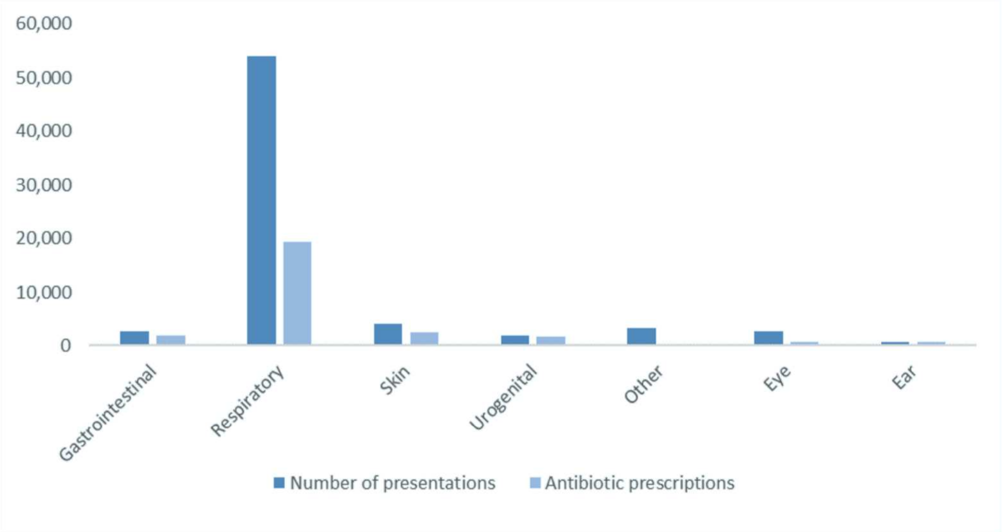

Figure 4: Number of acute presentations by single systems and whether antibiotics were prescribed

170x91mm (300 x 300 DPI)

Supplementary material

Antibiotic search list

- Amoxicillin
- Cefixime
- Ceftriaxone
- Cephalexin
- Ciprofloxacin
- Co-amoxiclav/ augmentin
- Co-trimoxazole/bactrim
- Dicloxacillin
- Doxycycline
- Erythromycin
- Metronidazole
- Norfloxacin
- Penicillin V
- Roxithromycin
- TC mycin/ tetracycline

Table S1: ICD 10 codes for infection used for the inclusion criteria

Code	Description	Excluded code (number)
**A00-B99**	Certain infectious and parasitic diseases	A15 (167), A16 (29), A18 (7), A31.9 (1), B18 (18), B24 (85), B85 (671)
**G00-G07**	Inflammatory diseases of the central nervous system	
**H00-01**	Hordeolum, chalazion and other inflammation of the eyelid	H01.1 (35)
**H05.0**	Acute inflammation of orbit	
**H10**	Conjunctivitis	
**H60-H70**	Otitis externa, otitis media and mastoiditis	H61 (112)
**H72-73**	Perforation and other disorders of the tympanic membrane	H73.9 (2)
**J00-43**	Respiratory tract infections	J30 (150), J31 (8), J33 (1), J35.1 (1)
**J47**	Bronchiectasis	
**K05**	Gingivitis and periodontal diseases	Exclude all (9,469)
**K11-12**	Diseases of salivary glands, stomatitis and related lesions	K11.1 (3), K11.88 (2), K11.9 (1), K12.0 (682), K12.1 (716)
**K35-37**	Appendicitis	
**K57**	Diverticulitis	K57 (2)

For peer review only

**K61**	Abscess of anal and rectal regions	
**K81**	Cholecystitis	
**K83-85**	Cholangitis and pancreatitis	
**L00-08**	Infections of the skin and subcutaneous tissue	
**L20-22**	Dermatitis	L20 (23), L21 (19), L22 (5)
**L30.3**	Infective dermatitis	
**L70-73.2**	Acne, rosacea follicular cysts and follicular disorders	
**M00-03**	Infectious arthropathies	M0013 (1), M0023 (1), M0167 (1), M020 (1)
**M60**	Myositis	M60.1-M6099 (3,604)
**N10-11**	Tubulo-interstitial nephritis	
**N30**	Cystitis	
**N34**	Urethritis and urethral syndrome	
**N39.0**	Urinary tract infection, site not specified	
**N41**	Inflammatory diseases of prostate	
**N45**	Orchitis and epididymitis	
**N48-49**	Other disorders of male genital organs	N48.9 (1)
**N61**	Inflammatory disorders of breast	
**N70-76**	Inflammatory diseases of female pelvic organs	
**O08.0**	Genital tract and pelvic infection following abortion and ectopic and molar pregnancy	
**O23**	Infections of genitourinary tract in pregnancy	
**O85-86**	Puerperal sepsis and other puerperal infections	
**P35-9**	Infections specific to the perinatal period	
**R05**	Cough	
**R11**	Nausea and vomiting	
**R30**	Pain associated with micturition	
**R36**	Urethral discharge	
**R50**	Fever	

For peer review only

Table S2: The number of initial presentations for each inclusion criteria and the percentage prescribed antibiotics during their illness episode

Age (years)	Number of presentations n/N (%)	Number of patients receiving an antibiotic prescription n/N (%)
**0-4**	18,073/83,659 (21.6)	6,110/18,073 (33.8)
**5-11**	13,775/83,659 (16.5)	6,318/13,775 (45.9)
**12-24**	10,533/83,659 (12.6)	5,888/10,533 (55.9)
**25-39**	11,025/83,659 (13.2)	6,167/11,025 (55.9)
**40-64**	23,134/83,659 (27.7)	11,843/23,134 (51.2)
**65 or over**	7,119/83,659 (8.5)	2,915/7,119 (41)
**Total**	83,659 (100)	39,241/83,659 (46.9)

Table S3: The number of presentations per age group and the percentage of each group prescribed an antibiotic

Inclusion criteria	Total initial presentations	Antibiotic prescription during the illness episode
**History of fever n/N (%)**	29,246/82,976 (35.3%)	11,725/29,246 (40.1%)
**Temperature >37.5°C n/N (%)**	10,508/76,644 (13.7%)	5,003/10,508 (47.6%)
**ICD 10 code for infection n/N (%)**	70,137/83,338 (84.2%)	27,234/70,137 (38.8%)
**Antibiotic prescription n/N (%)**	37,011/83,661 (44.2%)	39,242/83,661 (46.9%)

For peer review only

Diagnosis	Number of presentations n/N (%)	Number of antibiotic prescriptions during the illness episode n/N (%)
**Respiratory**		
Common cold	34,549/53,819 (64.2)	3,643/34,549 (10.5)
Acute sinusitis	30/53,819 (0.1)	25/30 (83.3)
Acute pharyngitis	13,080/53,819 (24.3)	11,607/13,080 (88.7)
Acute tonsillitis	3,459/53,819 (6.4)	3,014/3,459 (87.1)
Other URTIs	357/53,819 (0.7)	278/357 (77.9)
Acute LRTIs	663/53,819 (1.2)	541/663 (81.6)
Chronic bronchitis, emphysema & bronchiectasis	60/53,819 (0.1)	10/60 (16.7)
Cough	1,621/53,819 (3)	99/1,621 (6.1)
Sub total	53,819 (100)	19,217/53,819 (35.7)
**Gastrointestinal**		
Bacterial intestinal infections or intoxications	199/2,706 (7.4)	127/199 (63.8)
Viral enteritis	46/2,706 (1.7)	4/46 (8.7)
GE & colitis	2,412/2,706 (89.1)	1,614/2,412 (66.9)
Appendicitis	21/2,706 (0.8)	2/21 (9.5)
Other	9/2,706 (0.3)	2/9 (22.2)
Sialoadenitis	19/2,706 (0.7)	16/19 (84.2)
Sub total	2,706 (100)	1,765/2,706 (65.2)
**Skin**		
Infective dermatitis	85/4,060 (2.1)	70/85 (82.4)
Dermatophytosis	902/4,060 (22.2)	92/902 (10.2)
Other superficial mycoses	197/4,060 (4.9)	14/197 (7.1)
Candidiasis	101/4,060 (2.5)	23/101 (22.8)
Other	64/4,060 (1.6)	52/64 (81.3)
Scabies & infestations	52/4,060 (1.3)	8/52 (15.4)
Cellulitis & abscesses	841/4,060 (20.7)	618/841 (73.5)
Bacterial skin infections	533/4,060 (13.1)	464/533 (87.1)
Furuncles, caruncles & cysts	947/4,060 (23.3)	780/947 (82.4)
Other local infection of the skin & subcutaneous tissue	338/4,060 (8.3)	290/338 (85.8)
Sub total	4,060 (100)	2,411/4060 (59.4)
**Eye**		
Conjunctivitis	2,097/2,698 (77.7)	330/2,097 (15.7)
Hordeolum & chalazion	319/2,698 (11.8)	256/319 (80.3)
Other inflammation of the eyelid & orbit	268/2,698 (9.9)	98/268 (36.6)
Trachoma	14/2,698 (0.5)	5/14 (35.7)

For peer review only

Sub total	2,698 (100)	689/2,698 (25.5)
**Ear**		
Otitis externa	464/753 (61.6)	369/464 (79.5)
Otitis media	243/753 (32.3)	197/243 (81.1)
Mastoiditis	16/753 (2.1)	9/16 (56.3)
Perforation of tympanic membrane & other disorders	30/753 (4)	25/30 (83.3)
Sub total	753 (100)	600/753 (79.7)
**Urogenital**		
Acute tubulo-interstitial nephritis	36/1,871 (1.9)	32/36 (88.9)
Other	17/1,871 (0.9)	12/17 (70.6)
Cystitis, UTI, dysuria, urethritis & urethral syndrome	1,370/1,871 (73.2)	1,291/1,370 (94.2)
Other disorders of male genital organs	32/1,871 (1.7)	20/32 (62.5)
Other inflammatory disorders of female pelvic organs	148/1,871 (7.9)	115/148 (77.7)
Other inflammatory disorders of the vagina & vulva	268/1,871 (14.3)	149/268 (55.6)
Sub total	1,871 (100)	1,619/1,871 (86.5)
**Other**		
Bacterial	85/3,208 (2.7)	28/85 (32.9)
Unknown aetiology	33/3,208 (1)	14/33 (42.4)
Viral	728/3,208 (22.7)	153/728 (21)
Fungal	36/3,208 (1.1)	2/36 (5.6)
Protozoal	10/3,208 (0.3)	0/10 (0)
Parasitic	1,880/3,208 (58.6)	99/1,880 (5.3)
Nausea & vomiting	268/3,208 (8.4)	30/268 (11.2)
Fever of unknown or other origin	168/3,208 (5.2)	10/168 (6)
Sub total	3,208 (100)	336/3,208 (10.5)

Table S4: The number of presentations per diagnosis and system and whether antibiotics were prescribed for that illness episode

For peer review only

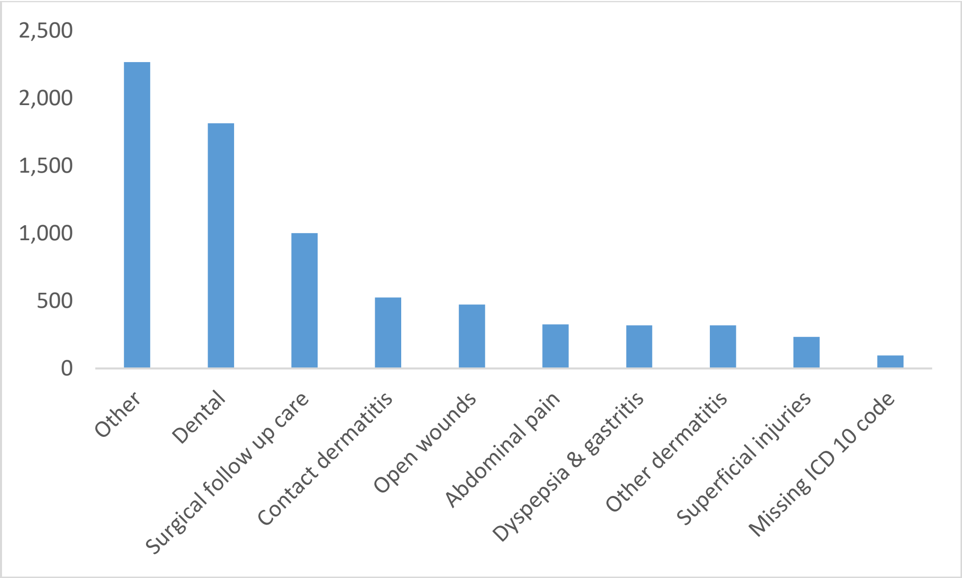

Figure S1: Single diagnoses used for antibiotic prescriptions without a history of fever, temperature or ICD 10 code for infection

For peer review only

ORD statement – checklist of items, extended from the STROBE statement, that should be reported in observational stud collected health data.

	Item No.	STROBE items	Location in manuscript where items are reported	RECORD items	Location manuscri where ite reported
**bstract**					
	1	(a) Indicate the study's design with a commonly used term in the title or the abstract (b) Provide in the abstract an informative and balanced summary of what was done and what was found		RECORD 1.1: The type of data used should be specified in the title or abstract. When possible, the name of the databases used should be included.<br><br>RECORD 1.2: If applicable, the geographic region and timeframe within which the study took place should be reported in the title or abstract.<br><br>RECORD 1.3: If linkage between databases was conducted for the study, this should be clearly stated in the title or abstract.	Abstract p 11<br><br>Abstract p 11&13<br><br>NA
**on**					
d	2	Explain the scientific background and rationale for the investigation being reported			Page 3 Backgrou section
	3	State specific objectives, including any prespecified hypotheses			Page 3, li Page 4, li 46: Study outcomes
gn	4	Present key elements of study design early in the paper			Page 4 lin
	5	Describe the setting, locations, and relevant dates, including periods of recruitment, exposure, follow-up, and data collection			Page 3 lin page 5 lin

For peer review only

s	6	*(a) Cohort study* - Give the eligibility criteria, and the sources and methods of selection of participants. Describe methods of follow-up<br>*Case-control study* - Give the eligibility criteria, and the sources and methods of case ascertainment and control selection. Give the rationale for the choice of cases and controls<br>*Cross-sectional study* - Give the eligibility criteria, and the sources and methods of selection of participants<br><br>*(b) Cohort study* - For matched studies, give matching criteria and number of exposed and unexposed<br>*Case-control study* - For matched studies, give matching criteria and the number of controls per case		RECORD 6.1: The methods of study population selection (such as codes or algorithms used to identify subjects) should be listed in detail. If this is not possible, an explanation should be provided.<br><br>RECORD 6.2: Any validation studies of the codes or algorithms used to select the population should be referenced. If validation was conducted for this study and not published elsewhere, detailed methods and results should be provided.<br><br>RECORD 6.3: If the study involved linkage of databases, consider use of a flow diagram or other graphical display to demonstrate the data linkage process, including the number of individuals with linked data at each stage.	Page 4 lin<br>48-page 5<br><br>NA<br><br>NA
	7	Clearly define all outcomes, exposures, predictors, potential confounders, and effect modifiers. Give diagnostic criteria, if applicable.		RECORD 7.1: A complete list of codes and algorithms used to classify exposures, outcomes, confounders, and effect modifiers should be provided. If these cannot be reported, an explanation should be provided.	Page 5 lin<br>suppleme<br>material
es/<br>nt	8	For each variable of interest, give sources of data and details of methods of assessment (measurement).<br>Describe comparability of assessment methods if there is more than one group			Page 5 lin<br>page 6 lin
	9	Describe any efforts to address			NA

For peer review only

		potential sources of bias			
	10	Explain how the study size was arrived at			NA
e	11	Explain how quantitative variables were handled in the analyses. If applicable, describe which groupings were chosen, and why			Page 5 lin<br>page 6 lin
	12	(a) Describe all statistical methods, including those used to control for confounding<br>(b) Describe any methods used to examine subgroups and interactions<br>(c) Explain how missing data were addressed<br>(d) *Cohort study* - If applicable, explain how loss to follow-up was addressed<br>*Case-control study* - If applicable, explain how matching of cases and controls was addressed<br>*Cross-sectional study* - If applicable, describe analytical methods taking account of sampling strategy<br>(e) Describe any sensitivity analyses			Page 5 lin<br>33- page [illegible]
s and<br>ethods		..		RECORD 12.1: Authors should describe the extent to which the investigators had access to the database population used to create the study population.<br><br>RECORD 12.2: Authors should provide information on the data cleaning methods used in the study.	Page 4 lin<br>53<br><br>Page 5 lin

For peer review only

					page 5 lin
		..		RECORD 12.3: State whether the study included person-level, institutional-level, or other data linkage across two or more databases. The methods of linkage and methods of linkage quality evaluation should be provided.	NA
s	13	(a) Report the numbers of individuals at each stage of the study (*e.g.*, numbers potentially eligible, examined for eligibility, confirmed eligible, included in the study, completing follow-up, and analysed)<br>(b) Give reasons for non-participation at each stage.<br>(c) Consider use of a flow diagram		RECORD 13.1: Describe in detail the selection of the persons included in the study (*i.e.,* study population selection) including filtering based on data quality, data availability and linkage. The selection of included persons can be described in the text and/or by means of the study flow diagram.	Page 4 lin 38<br>Page 6 lin<br>page 7 lin
e data	14	(a) Give characteristics of study participants (*e.g.*, demographic, clinical, social) and information on exposures and potential confounders<br>(b) Indicate the number of participants with missing data for each variable of interest<br>(c) *Cohort study* - summarise follow-up time (*e.g.*, average and total amount)			Page 7 lin<br>page 8 lin
ata	15	*Cohort study* - Report numbers of outcome events or summary measures over time<br>*Case-control study* - Report numbers in each exposure category, or summary measures of exposure			Page 7 lin 55

For peer review only

		Cross-sectional study - Report numbers of outcome events or summary measures			
s	16	(a) Give unadjusted estimates and, if applicable, confounder-adjusted estimates and their precision (e.g., 95% confidence interval). Make clear which confounders were adjusted for and why they were included<br>(b) Report category boundaries when continuous variables were categorized<br>(c) If relevant, consider translating estimates of relative risk into absolute risk for a meaningful time period			Page 7 lin page 8 lin
yses	17	Report other analyses done—e.g., analyses of subgroups and interactions, and sensitivity analyses			Page 8 lin page 9 lin
	18	Summarise key results with reference to study objectives			Page 9 lin 30
s	19	Discuss limitations of the study, taking into account sources of potential bias or imprecision. Discuss both direction and magnitude of any potential bias		RECORD 19.1: Discuss the implications of using data that were not created or collected to answer the specific research question(s). Include discussion of misclassification bias, unmeasured confounding, missing data, and changing eligibility over time, as they pertain to the study being reported.	Page 10 li 54, page 1 6-14
on	20	Give a cautious overall interpretation of results considering objectives, limitations, multiplicity of analyses, results from similar			Page 9 lin page 11 li

For peer review only

		studies, and other relevant evidence			
bility	21	Discuss the generalisability (external validity) of the study results			Page 10 li 44
**rmation**					
	22	Give the source of funding and the role of the funders for the present study and, if applicable, for the original study on which the present article is based			Page 11 li 44
ty of aw ng		..		RECORD 22.1: Authors should provide information on how to access any supplemental information such as the study protocol, raw data, or programming code.	Page 12 li 19

: Benchimol EI, Smeeth L, Guttmann A, Harron K, Moher D, Petersen I, Sørensen HT, von Elm E, Langan SM, the RECORD
. The REporting of studies Conducted using Observational Routinely-collected health Data (RECORD) Statement. *PLoS Medi*

is protected under Creative Commons Attribution (CC BY) license.

For peer review only

BMJ Open

A Retrospective Review of the Management of Acute Infections and the Indications for Antibiotic Prescription in Primary Care in Northern Thailand

Journal:	*BMJ Open*
Manuscript ID	bmjopen-2018-022250.R2
Article Type:	Research
Date Submitted by the Author:	08-Jun-2018

Complete List of Authors:	Greer, Rachel; Mahidol University, Mahidol-Oxford Tropical Medicine Research Unit, Faculty of Tropical Medicine; University of Oxford, Centre for Tropical Medicine and Global Health, Nuffield Department of Medicine,<br>Intralawan, Daranee; Chiangrai Regional Hospital, Social and Preventive Medicine Department<br>Mukaka, Mavuto; Mahidol University, Mahidol-Oxford Tropical Medicine Research Unit, Faculty of Tropical Medicine; University of Oxford, Centre for Tropical Medicine and Global Health, Nuffield Department of Medicine,<br>Wannapinij , Prapass; Mahidol University, Mahidol-Oxford Tropical Medicine Research Unit, Faculty of Tropical Medicine<br>Day, Nicholas; Mahidol University, Mahidol-Oxford Tropical Medicine Research Unit, Faculty of Tropical Medicine; University of Oxford, Centre for Tropical Medicine and Global Health, Nuffield Department of Medicine,<br>Nedsuwan, Supalert; Chiangrai Regional Hospital, Social and Preventive Medicine Department<br>Lubell, Yoel ; Mahidol University, Mahidol-Oxford Tropical Medicine Research Unit, Faculty of Tropical Medicine; University of Oxford, Centre for Tropical Medicine and Global Health, Nuffield Department of Medicine,

**Primary Subject Heading**:	Infectious diseases
Secondary Subject Heading:	General practice / Family practice
Keywords:	antibiotic use, PRIMARY CARE, Thailand, fever, Respiratory infections < THORACIC MEDICINE

SCHOLARONE™
Manuscripts

A RETROSPECTIVE REVIEW OF THE MANAGEMENT OF ACUTE INFECTIONS AND THE INDICATIONS FOR ANTIBIOTIC PRESCRIPTION IN PRIMARY CARE IN NORTHERN THAILAND

Rachel C. Greer[1, 2], Daranee Intralawan[3], Mavuto Mukaka[1, 2], Prapass Wannapinij[1], Nicholas P.J. Day[1, 2], Supalert Nedsuwan[3], Yoel Lubell[1, 2]

[1] Mahidol-Oxford Tropical Medicine Research Unit, Faculty of Tropical Medicine, Mahidol University, Bangkok, Thailand
[2] Centre for Tropical Medicine and Global Health, Nuffield Department of Medicine, University of Oxford, Oxford, United Kingdom
[3] Social and Preventive Medicine Department, Chiangrai Regional Hospital, Chiangrai, Thailand

Corresponding author

Rachel Claire Greer. Address: Mahidol-Oxford Tropical Medicine Research Unit, Faculty of Tropical Medicine, Mahidol University, 3rd Floor, 60th Anniversary Chalermprakiat Building, 420/6 Rajvithi Road, Ratchathewi District, Bangkok 10400, Thailand. Email: rachel@tropmedres.ac Phone: +66 52 029842

Word count: 4008

For peer review only

ABSTRACT

Introduction Antibiotic use in low and middle income countries continues to rise despite the knowledge that antibiotic overuse can lead to antimicrobial resistance. There is a paucity of detailed data on the use of antibiotics in primary care in low resource settings.

Objective Describe the presentation of acute infections and the indications for antibiotic prescription.

Design A two year retrospective review of routinely collected data.

Setting All 32 primary care units in one district in northern Thailand.

Participants Patients attending primary care with a history of fever, documented temperature, ICD 10 code for infection or prescribed a systemic antibiotic. Patients attending after the initiation of a study on C-reactive protein testing in four centres were excluded.

Outcome measures The proportion of patients prescribed an antibiotic and the frequency of clinical presentations.

Results 762,868 patients attended the health centers, of whom 103,196 met the inclusion criteria, 5,966 were excluded resulting in 97,230 attendances consisting of 83,661 illness episodes.

46.9% (39,242) of patients were prescribed an antibiotic during their illness. Indications for antibiotic prescription in the multivariable logistic regression analysis included male sex (aOR 1.21 [CI 1.16-1.28], p <0.001), adults (aOR 1.77 [CI 1.57-2, p <0.001]) and a temperature >37.5°C (aOR 1.24 [CI 1.03-1.48, p 0.020]). 77.9% of the presentations were for respiratory related problems, of which 98.6% were upper respiratory tract infections. The leading infection diagnoses were common cold (50%), acute pharyngitis (18.9%) and acute tonsillitis (5%), which were prescribed antibiotics in 10.5%, 88.7% and 87.1% of cases respectively. Amoxicillin was the most commonly prescribed antibiotic.

Conclusions Nearly half of the patients received an antibiotic, the majority of whom had a respiratory infection. The results can be used to plan interventions to improve the rational use of antibiotics. Further studies in private facilities, pharmacies and dental clinics are required.

Word count: 299

Keywords: antibiotic use, primary care, Thailand, fever, respiratory infections

ARTICLE SUMMARY

Strengths and limitations of this study

- Over 80,000 illness episodes reviewed from all primary care units in a district, over a two year time period
- Wide range of infections included rather than focusing on one specific infection
- Use of routine electronic data (no Hawthorne Effect), making this work reproducible
- Only included public healthcare facilities
- Reliant on the correct coding and clinical diagnoses of illnesses

For peer review only

BACKGROUND

The proportion of global deaths attributable to communicable diseases has greatly reduced in recent years. Despite these improvements, 10.6% of deaths worldwide in 2015 were thought to be caused by lower respiratory tract infections (LRTIs), diarrhoea, and tuberculosis (TB).[1] In under five year olds, 51.8% of deaths worldwide were due to infectious causes in 2013, with pneumonia causing 14.8% of the deaths overall.[2] In Thailand in 2010, respiratory infections were the leading cause of hospitalisations and deaths in children under the age of five.[3] Prompt access to appropriate antibiotics is vital to prevent many of these unnecessary deaths.[4] But while inappropriate or no treatment remains a clear cause for concern, the global antibiotic consumption rate increased by 39% between 2000 and 2015, fueled by low and middle income countries (LMIC),[5 6] with the majority of antibiotics being consumed in the community.[7]

Overuse and misuse of antibiotics have been linked to the development of antimicrobial resistance (AMR).[7-9] Antibiotics prescribed to individuals in primary care have been associated with bacterial resistance in that individual for up to 12 months, and longer and more frequent antibiotic courses are more likely to cause resistance.[10] The World Health Organization has described AMR in Southeast Asia as a 'burgeoning and often neglected' issue, stating that a 'post-antibiotic era' may become reality, resulting in common infections and minor injuries being untreatable.[11] In Thailand in 2010, there were an estimated 19,122 deaths attributable to multidrug resistant hospital-acquired infections.[12] Thailand has been making sustained efforts to reduce inappropriate antibiotic use; its Antibiotic Smart Use program started in 2007 and targets three conditions which are unlikely to require antibiotic treatment but for which they are commonly prescribed: upper respiratory tract infections (URTIs), acute diarrhoea and simple wounds.[13] Prescribing targets have been incorporated into the public health system's pay for performance criteria. In August 2016 the Thai government endorsed a national strategic plan for AMR which aims to optimize antimicrobial drug use and reduce the mean consumption of antimicrobials in humans by 20% by 2021.[14]

To appreciate the scale of the problem and to target future interventions, a greater understanding of the acute infections presenting to primary care and the conditions for which antibiotics are used in LMICs is required. Such data, however, are limited,[15] with most studies deriving their estimates from small samples of health providers and over a limited timeframe, therefore neglecting possible seasonal and spatial variation and other secular trends. In this paper we describe the proportion of patients receiving an antibiotic prescription and indications for antibiotic use in 32 primary care units (PCUs) across a district in northern Thailand over a two year period.

METHOD

A retrospective computerised search of routinely collected data from primary care units in Mueang Chiangrai District between January 2015 and December 2016 was carried out.

Study setting

Thailand is an upper-middle income country. In 2016 its gross domestic product (GDP) was 407 billion USD. The average life expectancy at birth is 75 years.[16] Chiangrai is the most northern province in Thailand and shares borders with Laos and Myanmar. It has a population of 1,282,544, of whom 241,436

For peer review only

reside in Mueang Chiangrai District.[17 18] Thailand has three seasons; the wet season typically runs from July to October, the cool season from November to February and the hot season from March to June.

Thailand's healthcare system is made up of public and private providers. Universal health coverage was established in 2002 following significant investment in the healthcare system and infrastructure since the 1970s. In rural and poorer areas primary healthcare is predominantly provided by the public healthcare system whereas in urban areas hospitals and private clinics play a larger role.[19]

Antibiotics can be bought directly from pharmacies and local stores as well as being prescribed by healthcare workers. Community antibiotic guidelines exist for some but not all common infections, including assessment criteria (e.g. the Centor criteria for acute tonsillitis), first line antibiotics, their dose and duration. There are prescribing restrictions in place for some broad spectrum antibiotics such as amoxicillin and clavulanic acid (Co-amoxiclav), which cannot be prescribed by nurses working in the public primary care system. More comprehensive hospital based guidelines are available.

In Mueang Chiangrai District family medicine doctors based at the provincial hospital oversee 32 public PCUs which are staffed primarily by two to five nurses and public health officers. On average PCUs look after 5000 patients each.[19] They provide care for acute and chronic conditions as well as providing preventative services such as immunisations, cervical screening and health education. Dental and traditional medicine services are also available. The furthest PCU is two hours' drive through the mountains from the provincial hospital in Chiangrai city (see figure 1). Finger-prick blood glucose tests are the only investigations routinely available on site.

Inclusion criteria

Patients were identified with at least one of the following:

- Systemic antibiotic prescription
- International Statistical Classification of Diseases (ICD) 10 code for infection (see supplementary material, table S1)
- Fever as the chief complaint
- Documented temperature >37.5°C at the PCU

We excluded patients attending PCUs used as study sites during or after a recent trial on the use of C-reactive protein (CRP) point of care tests (https://www.clinicaltrials.gov/ct2/show/NCT02758821?term=NCT02758821&rank=1).

Study outcomes

The primary outcome was the overall proportion of illness episodes prescribed an antibiotic. Risk factors for antibiotic use are reported as well as the percentages of patients receiving antibiotics according to their diagnosis, percentages of individual antibiotics used and the frequency and type of acute infection presentations.

Data collection

With the approval of the Chiangrai Provincial and Public Health Office (PHO), a research data manager accessed the PHO's routine medical records database to search for relevant patients and extract the

For peer review only

pre-specified variables. Data collected consisted of the PCU attended, patients' number, age, sex, date of visit, chief complaint, temperature, ICD 10 code, and drug prescriptions.

Data cleaning and coding

The inclusion criteria were classified as being present, absent or that the data were missing. Antibiotics were searched for in the prescription field (free text) and coded as prescribed (yes or no). A predefined antibiotic list (see supplementary material) was generated using the formulary. All medications prescribed in the dataset were reviewed to ensure no antibiotics were omitted due to spelling errors or their absence from our original list. If no prescription was recorded we made the assumption that this was because no medication was given rather than the data being missing.

Our predefined list of ICD 10 codes for infection (see supplementary material, table S1) were searched for in the diagnosis field (free text field, containing ICD 10 codes only) and then coded as present or absent. Our list of ICD 10 codes were reviewed with the other variables to ensure their appropriateness.

We searched for the word 'fever' in Thai in the chief complaint field (free text). In some cases 'no fever' was recorded, or the word 'fever' was present but part of a phrase alluding to a patient more generally, or 'influenza vaccine'. This field was checked manually with the help of two native northern Thai speaking study nurses. History of fever in the chief complaint was then coded as yes or no. Documented temperatures over 37.5°C at the PCU were searched for in the temperature field and then coded as temperature >37.5°C yes or no.

Repeat attendances within one month were classed as one illness episode allowing for the detection of subsequent antibiotics or treatment changes. All other indications such as the chief complaint and temperature were taken from the initial presentation. Children were defined as being under 12 years of age. The ICD 10 codes were grouped into gastrointestinal, respiratory, skin, urogenital, eye, ear and other categories. Each category was further broken down into conditions such as acute sinusitis and acute pharyngitis. The respiratory category was also grouped into upper and lower respiratory tract infections.

Statistical analyses

Descriptive statistics

Categorical variables were summarised using counts and percentages. Non-normally distributed data were described using medians and interquartile ranges and compared using the ranksum test. The proportions of patients prescribed an antibiotic in different demographic groups were summarised and compared using the chi-square test.

Logistic and Poisson regression models

A logistic regression model was used to model the binary outcome of antibiotic prescription (yes or no); both unadjusted and adjusted models were fitted and accounted for clustering of patients attending the same PCU. The odds ratios for the indications of antibiotic prescription were first obtained from univariate logistic regression models and then considered in a multivariable model if they had a p value of <0.05. Indications included sex, age category and documented temperature. The main purpose of this model was to identify risk factors that were independently associated with antibiotic prescription. A temperature of >37.5°C was used rather than the more subjective history of fever. ICD 10 codes were

For peer review only

not included because of their strong association with antibiotic prescriptions (e.g. a health worker's diagnosis of acute pharyngitis and its affiliated ICD 10 code was inherently associated with antibiotic prescription, as opposed to a diagnosis of 'common cold'). Furthermore a Poisson regression model of the monthly number of antibiotic prescriptions over the 24 month period was produced to obtain the Incidence Rate Ratios and 95% confidence intervals.

Time-series analysis

Monthly antibiotic prescriptions were weighted by the number of contributing PCUs per month and modelled over the two year period. When time-series analysis is used for forecasting, it is common to apply it to periods of five years or more, however, our aim was not to forecast into the future but to simply describe the current trends in antibiotic prescription.[20 21] We used a time-series analysis to separate long-term trends from seasonal variations.[22 23] Symmetric Locally Weighted Moving Averages (MA) were used. In this procedure, less weight was applied to time points (in months) furthest away from the present time point. The data was available on a monthly basis, however, a quarterly window was used to identify seasonality as follows: $\hat{X}_t = \frac{1}{9}(X_{t-2} + 2X_{t-1} + 3X_t + 2X_{t+1} + X_{t+2})$

Similarly a 12-month time-series window was used to obtain a trend line that would be sensitive to monthly changes but with reduced noise from seasonal variation:

$$\hat{X}_t = \frac{1}{24}(X_{t-6} + X_{t+6}) + \frac{1}{12}(X_t + X_{t-1} + X_{t+1} + X_{t-2} + X_{t+2} + X_{t-3} + X_{t+3} + X_{t-4} + X_{t+4} + X_{t-5} + X_{t+5})$$

Where $\hat{X}_t$ is the time-series modelled monthly prevalence of antibiotic prescription. Statistical significance was declared at alpha=0.05. Data analyses were performed with STATA version 14 (College Station, Texas, USA).

Ethical approval

Ethical approval was obtained from Chiangrai's Provincial and Public Health Office IRB (number 56/2560). Exemption was given by the Oxford Tropical Research Ethics Committee (OxTREC).

Patient and public involvement

Patients were not involved in the design of the study. Due to the study's retrospective nature patients were not involved in the recruitment processes. Study results will be disseminated through community presentations as well as educational updates for the healthcare workers and community volunteers.

RESULTS

762,868 patients attended the PCUs between the 1st of January 2015 and the 31st of December 2016. The majority of patients' attendances included a chronic disease review or screening, the most common

For peer review only

being screening for diseases such as diabetes, hypertension, mental health and dental disorders (145,410), essential hypertension reviews (98,822) and routine child health examinations (75,701).

The appropriateness of the ICD 10 codes for infection used in our inclusion criteria were reviewed alongside the other variables. For example, we found that patients with TB, HIV and Hepatitis B were only attending for regular medications rather than for acute illnesses so they were removed from the ICD 10 inclusion list. Mass head lice treatment at schools is carried out by the PCUs so these codes were also removed. The ICD 10 code 'K05' (dental) was also removed because it transpired that these patients are seen by dentists or dental nurses at the PCUs rather than by the regular PCU staff. All ICD 10 codes for myositis were removed from the inclusion criteria apart from M60.0 (infective myositis) because the other codes were being used for muscle pain or myalgia (see supplementary material, table S1).

In total 103,196 attendances met our inclusion criteria; 5,966 were then excluded because the PCUs they attended were involved in the CRP study before or during their attendance, resulting in 97,230 attendances (12.7%) meeting our inclusion and exclusion criteria. 13,569 repeat attendances within one month were classed as a single illness episode, leaving 83,661 illness episodes.

Patient characteristics

The median age was 24 years old with an interquartile range of 6 to 51 years old. Two patients had no age recorded. 54.7% of the patients were female (45,779) compared to 45.3% males (37,882) ($p<0.001$).

The proportion of patients meeting each inclusion criterion is shown in figure 2 and supplementary material table S2. 29,246 (35.3%) patients presented with a history of fever, while 10,508 (13.7%) had a temperature of more than 37.5°C at presentation. 8,871 (11.6%) patients had both a history of fever and a temperature.

Antibiotics

Medications were prescribed for 81,691 (97.7%) illness episodes. 37,011 (44.2%) patients were prescribed an antibiotic during their first visit, and this increased to 39,242 (46.9%) throughout their illness episodes.

Antibiotics were prescribed to:

- 49.2% of men compared to 45% of women ($p<0.001$)
- 39% of children compared to 51.8% of adults ($p<0.001$)
- 40.1% of those with a history of fever
- 47.6% with a temperature >37.5°C
- 38.8% with an ICD 10 code for infection

The proportion of patients within each age group prescribed an antibiotic varied, with the lowest rates in young children (0-4 year olds, 33.8%), peaking in adults (12-39 year olds, 55.9%) and then diminishing in the elderly (aged 65 years and older, 41%, see supplementary material, table S3).

The odds ratios for the univariate and multivariable logistic regression analyses are shown in table 1. All variables entered into the univariate model were significant so were added to the multivariable analysis. Indications for antibiotic prescription in the adjusted multivariable logistic regression analysis were male sex (aOR 1.21 [CI 1.16-1.28], $p<0.001$), patients aged 12 years of age or older (compared to those less

For peer review only

than 12 years old) (aOR 1.77 [CI 1.57-2, p <0.001]) and having a temperature of more than 37.5°C (aOR 1.24 [CI 1.03-1.48, p 0.02]).

Variable	OR (confidence interval)	p value	aOR (confidence interval)	p value
**Univariate analysis**			**Multivariable analysis**	
**Male sex**	1.18 (1.12-1.25)	<0.001	1.21 (1.16-1.28)	<0.001
**Aged ≥ 12 years old**	1.68 (1.48-1.90)	<0.001	1.77 (1.57-2)	<0.001
**Temperature >37.5°C**	1.05 (0.85-1.30)	0.197	1.24 (1.03-1.48)	0.020

Table 1: Univariate and multivariable logistic regression analyses accounting for clustering of patients attending the same PCU, showing all included variables and their association with antibiotic prescription

Figure 3 is a time series plot for the monthly prevalence of antibiotic prescriptions. Overall there was no significant trend; incidence rate ratio (IRR) =0.99, 95% CI (0.990, 1.007), p=0.796, although there is a suggestion of a downward trend beginning in the final 6 months The monthly prevalence of antibiotic prescriptions was at least 39% throughout the two year period. Patients attending in the wet season (July-October) were more likely to receive antibiotics (47.4%) than those attending in the hot and cold seasons (46.6%) p value 0.029. Overall prescription rates varied greatly between the PCUs from 8 to 71.6%, with prescribing consistently higher in adults than in children.

The majority of patients prescribed an antibiotic received amoxicillin (56.7%) or dicloxacillin (25.1%). Other antibiotics prescribed include norfloxacin (8.9%), co-trimoxazole (4.2%), penicillin V (1.2%), roxithromycin (1.2%), metronidazole (1.2%), erythromycin (0.7%), cephalexin (0.4%) and tetracycline (0.2%).

Presentations and antibiotic prescriptions

The number of acute presentations with ICD 10 codes for infection related to a single system are shown in figure 4. 77.9% of these presentations were for respiratory related problems. 98.6% of these were diagnosed with an URTI, 1.1% with an acute LRTI and 0.3% with a chronic LRTI, of these 36.1%, 81.8% and 53.5% were prescribed antibiotics respectively. The most common single infection diagnoses were common cold (34,549, 50%), acute pharyngitis (13,080, 18.9%) and acute tonsillitis (3,459, 5%), antibiotics were prescribed to 10.5%, 88.7% and 87.1% of the cases respectively (see table 2).

Diagnosis	Number of presentations n/N (%)	Episode antibiotics prescribed n/N (%)	Commonest antibiotic prescribed (%)
**Common cold**	34,549/69,115 (50)	3,643/34,549 (10.5)	Amoxicillin (71.7)
**Acute pharyngitis**	13,080/69,115 (18.9)	11,607/13,080 (88.7)	Amoxicillin (91.5)
**Acute tonsillitis**	3,459/69,115 (5)	3,014/3,459 (87.1)	Amoxicillin (93.4)
**Gastroenteritis & colitis unspecified**	2,412/69,115 (3.5)	1,614/2,412 (66.9)	Norfloxacin (68.8)
**Conjunctivitis**	2,097/69,115 (3.0)	330/2,097 (15.7)	Amoxicillin (56.4)
**Other helminthiases**	1,231/69,115 (1.8)	65/1,231 (5.3)	Amoxicillin (41.5)
**Cystitis**	1,230/69,115 (1.8)	1,165/1,230 (94.7)	Norfloxacin (75.9)

For peer review only

Table 2: Common diagnoses in patients with one single ICD 10 code for infection, whether antibiotics were prescribed and which antibiotic was mostly commonly used

Supplementary table S4 shows the number of individual infection diagnoses by systems and the rates of antibiotic prescriptions. Antibiotics were prescribed to 59.4% of skin infections, 81.1% of otitis media, 79.5% of otitis externa, 94.7% of cystitis, 80.3% of hordeolum (styes) and chalazions as well as 15.7% of conjunctivitis cases. Of the total antibiotics prescribed almost a third (29.6%) were given to those with acute pharyngitis, followed by common cold (9.3%), acute tonsillitis (7.7%), gastroenteritis and colitis (4.1%) and cystitis (3%) as the single infection diagnoses.

13.8% of patients (11,547) were prescribed antibiotics without a temperature, history of fever or ICD 10 code for infection. Of those who had a single ICD 10 code recorded 1,815 (24.6%) of these antibiotics were for dental reasons, 1,002 (13.6%) for surgical follow up care, 526 (7.1%) for contact dermatitis and 473 (6.4%) for open wounds (see supplementary material, figure S1). These patients were more likely to be male (54.3%, p value <0.001) and older (median age of 41 compared to 24 years old) than the main patient group.

DISCUSSION

To the best of our knowledge this is the largest review of acute illness presentations and community antibiotic prescribing in a LMIC. Over a two year period there were more than 97,000 attendances to 32 PCUs for acute infections and nearly half these patients received an antibiotic, with no significant change in prescribing levels over the two year study period. Studies of this magnitude are required to increase our knowledge of the scale of antibiotic prescribing as well as the common conditions they are used for.[24 25] Thailand's 2016 national strategic plan on AMR also highlighted the importance of monitoring and reporting antimicrobial consumption.[26]

Comparing overall antibiotic prescribing rates with other studies is challenging because of varying definitions of acute illnesses and the different patient populations. However the antibiotic prescribing rate in our study is more than double the prescribing in a Malaysian study but similar to studies in India and Laos.[27-29] A third of our patients had a history of fever, which is similar to a point prevalence study in India where fever was the most common symptom.[30] Almost 80% of the ICD 10 codes for infection were related to respiratory infections, which is consistent with respiratory infections being the leading cause of hospitalisations and deaths in Thai under five year olds[3] but is higher than the proportion of respiratory presentations in other South and Southeast Asian countries.[30 31] Antibiotic prescribing in Thailand for tonsillitis and pharyngitis remains high despite Group A beta-haemolytic Streptococci being isolated in only 3.8-7.9% of those with URTI.[32 33]

In the first phase of Thailand's Antibiotic Smart Use program overall antibiotic use in PCUs was reduced by between 39% and 46%. Prescriptions for the three target conditions (URTI, acute diarrhoea and simple wounds) reduced from 54.5% to 25.4%.[34] Despite the lower prescribing levels of 10.5% for common colds in our review, there were still 3,643 antibiotic prescriptions for this condition, alongside 88.7% of those with acute pharyngitis, 87.1% with acute tonsillitis and 66.9% with gastroenteritis and colitis receiving antibiotics; this is likely to represent the overuse of antibiotics. Open wounds and superficial injuries were common diagnoses in those prescribed an antibiotic without a history of fever,

For peer review only

temperature, or ICD 10 code for infection. The results reveal the ongoing high levels of prescribing for these conditions and present an opportunity to further reduce antibiotic use. Since late 2016 an antibiotic prescribing target of less than 20% for these three conditions has been incorporated into Thailand's rational drug use service plan as well as the pay for performance health criteria, and financial incentives are given to the PCUs achieving this target. A review of the long term effectiveness of this policy including any impact on patient safety is required.

Our study also identifies high levels of prescribing for skin infections, otitis media, otitis externa, cystitis, hordeolum (styes) and chalazions. A lack of available topical antibiotics may account for the high prescribing for skin infections as well as for otitis externa. However, despite antibacterial eye drops being available, 15.7% of conjunctivitis cases were still prescribed a systemic antibiotic. Urine dipstick tests are not available on site to assess patients with cystitis or suspected urinary tract infections. Introduction of these simple tests may help to rationalize prescribing for these conditions in a setting where urine cultures are not readily available or achievable.

While we did not set out to review dental prescribing, this area accounted for 25% of the antibiotics prescribed to those without a history of fever, temperature or ICD 10 code for infection, which warrants further investigation.

Some of the variation in antibiotic prescribing rates between PCUs may be accounted for by the degree of staff training. Two out of the three highest prescribing PCUs are staffed only by public health officers. The study findings are being used to guide educational updates and training for the PCU staff, with priority being given to those PCUs without nurses and with high prescription rates for conditions unlikely to require antibiotics.

A wide range of antibiotics are prescribed in the PCUs. Restrictions are in place for some broad-spectrum antibiotics such as amoxicillin and clavulanic acid (Co-amoxiclav) which cannot be prescribed. One area of concern is that less than 1% of the antibiotics being prescribed have activity against scrub typhus, which is the leading cause of hospital admission with acute undifferentiated fever in this region.[35]

Strengths and limitations

The main strength of this study is the large number of illness episodes included. The two year time period should allow for seasonal variations and disease epidemics. We reviewed prescribing in all of the PCUs in Mueang Chiangrai District which covers a large geographical area and has a range of rural and urban facilities, making the results generalizable to the region more broadly. Many studies have focused on prescribing for specific conditions such as URTIs but our study covers a wide range of infections that present in the community. Having research staff on site has been shown to influence healthcare workers' prescribing habits (the Hawthorne Effect), but due to the retrospective nature of the study this was not a source of bias. The use of routinely collected data means that this methodology could be repeated in other districts and provinces in Thailand, although a lot of the data are entered as free text which presents challenges for analysis. Searching for patients with a history of fever, for instance, was problematic because the Thai word 'ไข้' or fever is also part of the Thai words for patient, influenza, anti-pyrexials, etc.

For peer review only

Limitations of this study are that we only included public PCUs and have no data on antibiotic use by private clinics, pharmacies or family medicine doctors based at the provincial hospital, which requires further study. The PCU data is taken from routine electronic records and in some instances there were tranches of missing data (five PCUs had no recorded data for several months). Verifying the quality of some data is also challenging; coding of clinical diagnoses for instance using ICD 10 could be inconsistent between healthcare workers and in primary care the majority of infections are diagnosed clinically without any laboratory tests. However we used data from a subsample of patients enrolled in a clinical trial in four PCUs and compared them to their respective routine medical records. While minor discrepancies were found in their precise age and temperature the diagnoses and antibiotic prescribing data were consistent. Our decision to class all attendances within a one month period as a single illness episode means that we may have incorrectly classed some new illnesses as a repeat attendance but did allow us to review antibiotic prescribing over the course of the illness. The time series analysis was carried out using data from a two year time period, more definitive conclusions and trends may have become apparent if further time points and data were available.

CONCLUSIONS

This study provides much needed insight into the use of antibiotics in primary care in northern Thailand, allowing targeting of interventions to improve the rational use of antibiotics. Nearly half of all patients attending with an acute illness received an antibiotic. The majority of presentations were for respiratory infections. Further education and resources are required to support clinicians in the targeting of antibiotics. This could include the introduction of clinical algorithms and point of care tests such as CRP and urine dipsticks. Antibiotic guidelines are required for common conditions seen in primary care outside of the current Antibiotic Smart Use policy. Further studies including qualitative work are required to appreciate the use of antibiotics in other settings such as private facilities, pharmacies and dental clinics.

Funding statement

This research received no specific grant from any funding agency in the public, commercial or not-for-profit sectors. This study was part of the Wellcome Trust Major Overseas Programme in SE Asia (grant number 106698/Z/14/Z).

Competing interests

None declared

Author contribution statement

All authors were involved in the design of the study. PW collected the data. RCG carried out the analysis with support from YL. MM provided statistical support. RCG, YL, DI, SN interpreted the data. RCG wrote

For peer review only

the first draft of the paper. YL, NPJD and MM reviewed subsequent drafts. All authors approved the final draft for publication.

Acknowledgements

We would like to thank Wasuphal Kanjana for his help with data collection, Nipaphan Kanthawang and Pratakpong Wongkiti for their help with data cleaning and translation of the Thai abstract and Areerat Thaiprakhong for her assistance with figure 1.

Data sharing statement

We are unable to share additional unpublished data which falls under the jurisdiction of the Chiang Rai PHO.

Ethical approval

Ethical approval was obtained from Chiangrai's Provincial and Public Health Office IRB (number 56/2560). Exemption was given by the Oxford Tropical Research Ethics Committee (OxTREC). Individual informed consent was not required.

REFERENCES

1. The World Health Organization. The top 10 causes of death 2017 [Available from: http://www.who.int/mediacentre/factsheets/fs310/en/ (Accessed January 2018)
2. Liu L, Oza S, Hogan D, et al. Global, regional, and national causes of child mortality in 2000-13, with projections to inform post-2015 priorities: an updated systematic analysis. *Lancet* 2015;385(9966):430-40. doi: 10.1016/S0140-6736(14)61698-6 [published Online First: 2014/10/05]
3. Teeratakulpisarn J, Uppala R, Thepsuthammarat K, et al. Burden of Acute Lower Respiratory Infection in Children in Thailand in 2010: Have We Achieved the National Target in Under-five Morbidity and Mortality? *Journal of the Medical Association of Thailand = Chotmaihet thangphaet* 2012;95(7):S87-96.
4. Laxminarayan R, Matsoso P, Pant S, et al. Access to effective antimicrobials: a worldwide challenge. *Lancet* doi: 10.1016/S0140-6736(15)00474-2
5. Klein EY, Van Boeckel TP, Martinez EM, et al. Global increase and geographic convergence in antibiotic consumption between 2000 and 2015. *Proceedings of the National Academy of Sciences* 2018
6. Van Boeckel TP, Gandra S, Ashok A, et al. Global antibiotic consumption 2000 to 2010: an analysis of national pharmaceutical sales data. *Lancet Infect Dis*;14(8):742-50. doi: 10.1016/S1473-3099(14)70780-7
7. The European Centre for Disease Prevention and Control (ECDC). Surveillance of antimicrobial consumption in Europe 2012. 2014 [Available from: https://ecdc.europa.eu/sites/portal/files/media/en/publications/Publications/antimicrobial-consumption-europe-esac-net-2012.pdf (Accessed August 2017)

For peer review only

8. Laxminarayan R, Duse A, Wattal C, et al. Antibiotic resistance - the need for global solutions. *Lancet Infect Dis* 2013;13(12):1057-98. doi: 10.1016/S1473-3099(13)70318-9
9. Gelband H, Miller-Petrie M, Pant S, et al. The State of the World's Antibiotics: Center for Disease Dynamics, Ecomomics & Policy (CDDEP). 2015 [Available from: https://www.cddep.org/publications/state_worlds_antibiotics_2015/ (Accessed October 2017)
10. Costelloe C, Metcalfe C, Lovering A, et al. Effect of antibiotic prescribing in primary care on antimicrobial resistance in individual patients: systematic review and meta-analysis. *BMJ* 2010;340 doi: 10.1136/bmj.c2096
11. The World Health Organization. Antimicrobial Resistance. Global Report on Surveillance 2014 [Available from: http://apps.who.int/iris/bitstream/10665/112642/1/9789241564748_eng.pdf?ua=1 (Accessed March 2017)
12. Lim C, Takahashi E, Hongsuwan M, et al. Epidemiology and burden of multidrug-resistant bacterial infection in a developing country. *eLife* 2016;5:e18082. doi: 10.7554/eLife.18082
13. Sumpradit N, Chongtrakul P, Anuwong K, et al. Antibiotics Smart Use: a workable model for promoting the rational use of medicines in Thailand. *Bulletin of the World Health Organization* 2012;90(12):905-13. doi: 10.2471/BLT.12.105445
14. Tangcharoensathien V, Sattayawutthipong W, Kanjanapimai S, et al. Antimicrobial resistance: from global agenda to national strategic plan, Thailand. *Bulletin of the World Health Organization* 2017;95(8):599-603. doi: 10.2471/BLT.16.179648
15. Zellweger RM, Carrique-Mas J, Limmathurotsakul D, et al. A current perspective on antimicrobial resistance in Southeast Asia. *J Antimicrob Chemother* 2017;72(11):2963-72. doi: 10.1093/jac/dkx260
16. World bank group. Thailand data 2017 [Available from: https://data.worldbank.org/country/thailand (Accessed October 2017)
17. National Statistical Office. Chiang Rai provincial statistical report 2017 [Available from: http://chiangrai.nso.go.th/images/data/%2060.compressed.pdf (Accessed April 2018)
18. Department of Provincial Administration. Official Statistics Registration Systems, 2016. [Available from: http://stat.dopa.go.th/stat/statnew/upstat_age.php] (Accessed November 2017)
19. Asia Pacific Observatory on Health Systems and Policies. The Kingdom of Thailand Health System Review. *Health Systems in Transition* 2015;5(5)
20. Albu JB, Sohler N, Li R, et al. An Interrupted Time Series Analysis to Determine the Effect of an Electronic Health Record–Based Intervention on Appropriate Screening for Type 2 Diabetes in Urban Primary Care Clinics in New York City. *Diabetes Care* 2017;40(8):1058.
21. Girgis A, Kelly B, Boyes A, et al. The PACT study protocol: a time series study investigating the impact, acceptability and cost of an integrated model for psychosocial screening, care and treatment of patients with urological and head and neck cancers. *BMJ Open* 2014;4(1)
22. Moineddin R, Upshur RE, Crighton E, et al. Autoregression as a means of assessing the strength of seasonality in a time series. *Popul Health Metr* 2003;1(1):10. doi: 10.1186/1478-7954-1-10 [published Online First: 2003/12/17]
23. Wall EC, Everett DB, Mukaka M, et al. Bacterial Meningitis in Malawian Adults, Adolescents, and Children During the Era of Antiretroviral Scale-up and Haemophilus influenzae Type b Vaccination, 2000–2012. *Clin Infect Dis* 2014;58(10):e137-e45. doi: 10.1093/cid/ciu057
24. O'Brien K, Bellis TW, Kelson M, et al. Clinical predictors of antibiotic prescribing for acutely ill children in primary care: an observational study. *Br J Gen Pract* 2015;65(638):e585.
25. Petersen I, Hayward AC. Antibacterial prescribing in primary care. *J Antimicrob Chemother* 2007;60(suppl_1):i43-i47. doi: 10.1093/jac/dkm156

For peer review only

26. Sumpradit N, Wongkongkathep S, Poonpolsup S, et al. New chapter in tackling antimicrobial resistance in Thailand. *BMJ* 2017;358
27. Keohavong B, Syhakhang L, Sengaloundeth S, et al. Rational use of drugs: prescribing and dispensing practices at public health facilities in Lao PDR. *Pharmacoepidemiology and Drug Saf* 2006;15(5):344-47. doi: 10.1002/pds.1169
28. Ab Rahman N, Teng CL, Sivasampu S. Antibiotic prescribing in public and private practice: a cross-sectional study in primary care clinics in Malaysia. *BMC Infect Dis* 2016;16 doi: 10.1186/s12879-016-1530-2
29. Kotwani A, Holloway K. Trends in antibiotic use among outpatients in New Delhi, India. *BMC Infect Dis* 2011;11:99-99. doi: 10.1186/1471-2334-11-99
30. Salvi S, Apte K, Madas S, et al. Symptoms and medical conditions in 204912 patients visiting primary health-care practitioners in India: a 1-day point prevalence study (the POSEIDON study). *Lancet Glob Health* 2015;3(12):e776-e84. doi: 10.1016/S2214-109X(15)00152-7
31. Mimi O, Tong SF, Nordin S, et al. A Comparison of Morbidity Patterns in Public and Private Primary Care Clinics in Malaysia. *Malaysian Family Physician : the Official Journal of the Academy of Family Physicians of Malaysia* 2011;6(1):19-25.
32. Boonyasiri A, Thamlikitkul V. Effectiveness of multifaceted interventions on rational use of antibiotics for patients with upper respiratory tract infections and acute diarrhea. *Journal of the Medical Association of Thailand = Chotmaihet thangphaet* 2014;97(3 SUPPL. 3):S13-S19.
33. Treebupachatsakul P, Tiengrim S, Thamlikitkul V. Upper respiratory tract infection in Thai adults: prevalence and prediction of bacterial causes, and effectiveness of using clinical practice guidelines. *Journal of the Medical Association of Thailand = Chotmaihet thangphaet* 2006;89(8):1178-86.
34. The World Health Organization. The Pursuit of Responsible Use of Medicine: Sharing and Learning from Country Experiences. 2012 [Available from:http://www.who.int/medicines/publications/responsible_use/en/] (Accessed December 2017)
35. Wangrangsimakul T, Althaus T, Mukaka M, et al. Causes of acute undifferentiated fever and the utility of biomarkers in Chiangrai, northern Thailand. *PLOS Negl Trop Dis* 2018;12(5):e0006477. doi: 10.1371/journal.pntd.0006477

Figure 1: Chiangrai and the 32 PCUs

Figure 2: A Venn diagram to show the inclusion criteria

Figure 3: Trend and seasonality of antibiotic prescriptions overlaid by mean antibiotic prescription rates per PCU

Figure 4: Number of acute presentations by single systems and whether antibiotics were prescribed

For peer review only

For peer review only

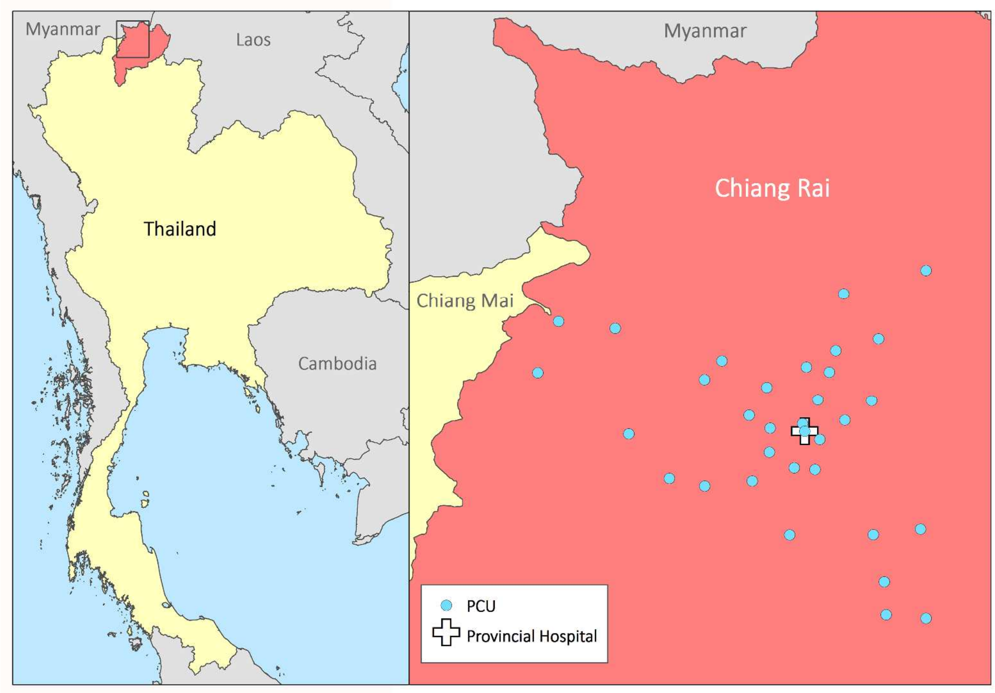

Figure 1: Chiangrai and the 32 PCUs

354x246mm (300 x 300 DPI)

For peer review only

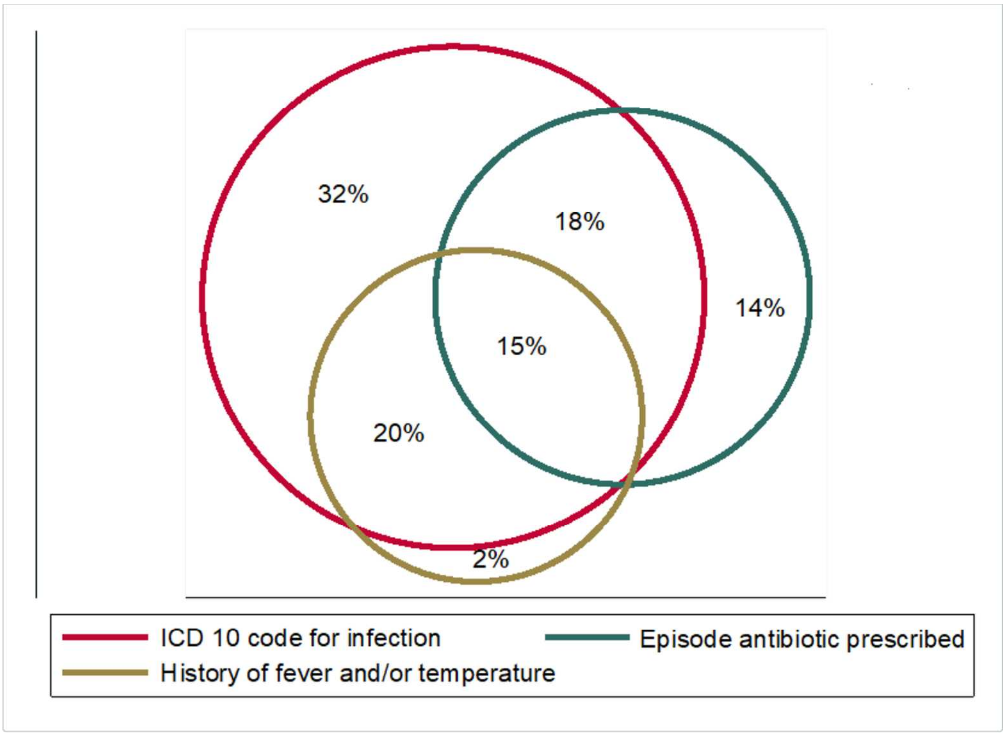

Figure 2: A Venn diagram to show the inclusion criteria

150x109mm (300 x 300 DPI)

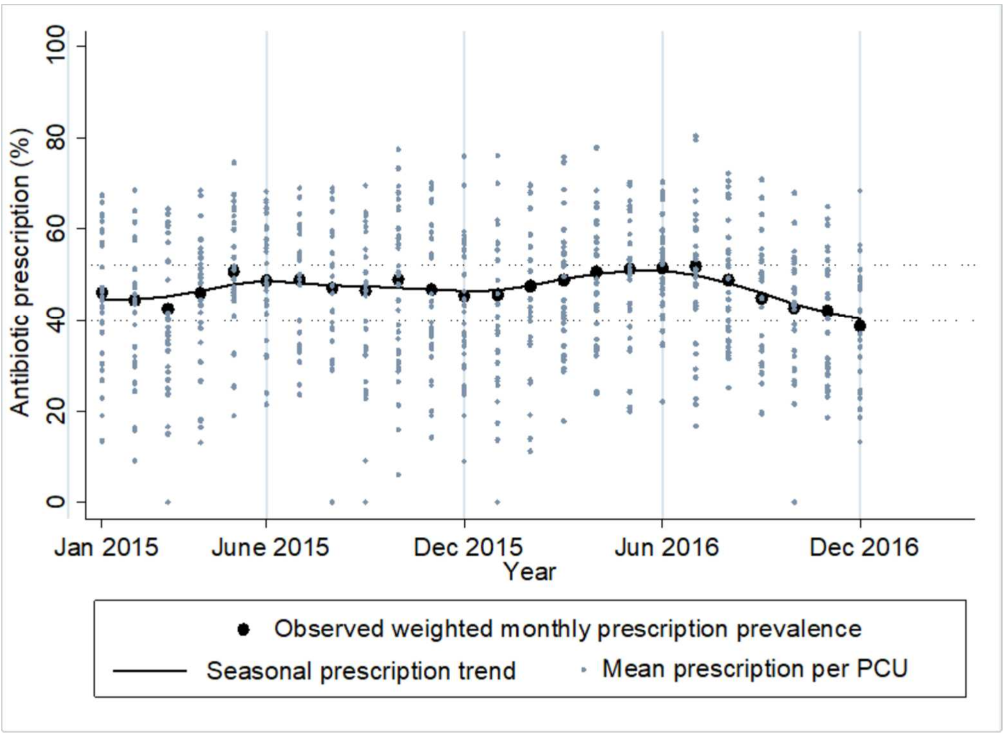

Figure 3: Trend and seasonality of antibiotic prescriptions overlaid by mean antibiotic prescription rates per PCU

127x92mm (300 x 300 DPI)

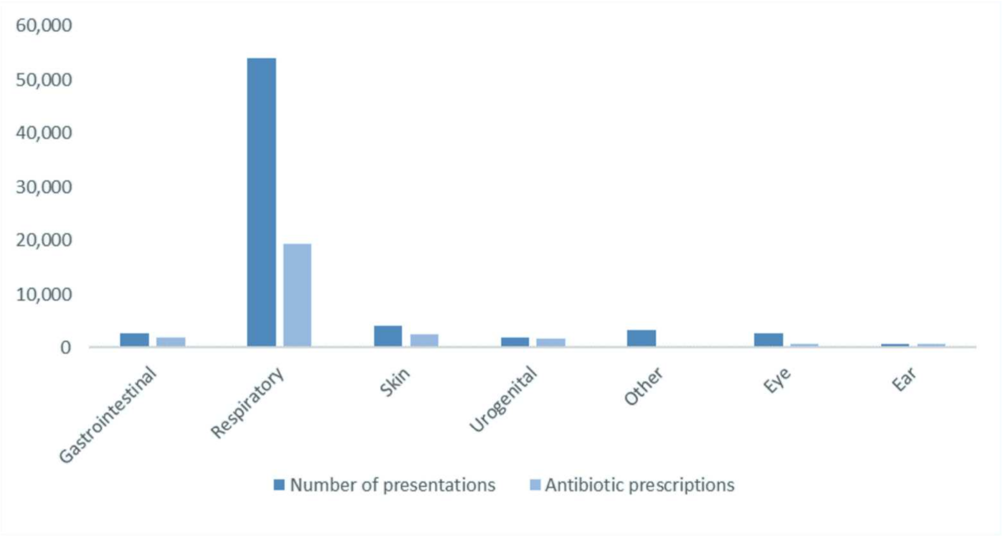

Figure 4: Number of acute presentations by single systems and whether antibiotics were prescribed

170x91mm (300 x 300 DPI)

For peer review only

Supplementary material

Antibiotic search list

- Amoxicillin
- Cefixime
- Ceftriaxone
- Cephalexin
- Ciprofloxacin
- Co-amoxiclav/ augmentin
- Co-trimoxazole/bactrim
- Dicloxacillin
- Doxycycline
- Erythromycin
- Metronidazole
- Norfloxacin
- Penicillin V
- Roxithromycin
- TC mycin/ tetracycline

Table S1: ICD 10 codes for infection used for the inclusion criteria

Code	Description	Excluded code (number)
**A00-B99**	Certain infectious and parasitic diseases	A15 (167), A16 (29), A18 (7), A31.9 (1), B18 (18), B24 (85), B85 (671)
**G00-G07**	Inflammatory diseases of the central nervous system	
**H00-01**	Hordeolum, chalazion and other inflammation of the eyelid	H01.1 (35)
**H05.0**	Acute inflammation of orbit	
**H10**	Conjunctivitis	
**H60-H70**	Otitis externa, otitis media and mastoiditis	H61 (112)
**H72-73**	Perforation and other disorders of the tympanic membrane	H73.9 (2)
**J00-43**	Respiratory tract infections	J30 (150), J31 (8), J33 (1), J35.1 (1)
**J47**	Bronchiectasis	
**K05**	Gingivitis and periodontal diseases	Exclude all (9,469)
**K11-12**	Diseases of salivary glands, stomatitis and related lesions	K11.1 (3), K11.88 (2), K11.9 (1), K12.0 (682), K12.1 (716)
**K35-37**	Appendicitis	
**K57**	Diverticulitis	K57 (2)

For peer review only

**K61**	Abscess of anal and rectal regions	
**K81**	Cholecystitis	
**K83-85**	Cholangitis and pancreatitis	
**L00-08**	Infections of the skin and subcutaneous tissue	
**L20-22**	Dermatitis	L20 (23), L21 (19), L22 (5)
**L30.3**	Infective dermatitis	
**L70-73.2**	Acne, rosacea follicular cysts and follicular disorders	
**M00-03**	Infectious arthropathies	M0013 (1), M0023 (1), M0167 (1), M020 (1)
**M60**	Myositis	M60.1-M6099 (3,604)
**N10-11**	Tubulo-interstitial nephritis	
**N30**	Cystitis	
**N34**	Urethritis and urethral syndrome	
**N39.0**	Urinary tract infection, site not specified	
**N41**	Inflammatory diseases of prostate	
**N45**	Orchitis and epididymitis	
**N48-49**	Other disorders of male genital organs	N48.9 (1)
**N61**	Inflammatory disorders of breast	
**N70-76**	Inflammatory diseases of female pelvic organs	
**O08.0**	Genital tract and pelvic infection following abortion and ectopic and molar pregnancy	
**O23**	Infections of genitourinary tract in pregnancy	
**O85-86**	Puerperal sepsis and other puerperal infections	
**P35-9**	Infections specific to the perinatal period	
**R05**	Cough	
**R11**	Nausea and vomiting	
**R30**	Pain associated with micturition	
**R36**	Urethral discharge	
**R50**	Fever	

For peer review only

Table S2: The number of initial presentations for each inclusion criteria and the percentage prescribed antibiotics during their illness episode

Age (years)	Number of presentations n/N (%)	Number of patients receiving an antibiotic prescription n/N (%)
**0-4**	18,073/83,659 (21.6)	6,110/18,073 (33.8)
**5-11**	13,775/83,659 (16.5)	6,318/13,775 (45.9)
**12-24**	10,533/83,659 (12.6)	5,888/10,533 (55.9)
**25-39**	11,025/83,659 (13.2)	6,167/11,025 (55.9)
**40-64**	23,134/83,659 (27.7)	11,843/23,134 (51.2)
**65 or over**	7,119/83,659 (8.5)	2,915/7,119 (41)
**Total**	83,659 (100)	39,241/83,659 (46.9)

Table S3: The number of presentations per age group and the percentage of each group prescribed an antibiotic

Inclusion criteria	Total initial presentations	Antibiotic prescription during the illness episode
**History of fever n/N (%)**	29,246/82,976 (35.3%)	11,725/29,246 (40.1%)
**Temperature >37.5°C n/N (%)**	10,508/76,644 (13.7%)	5,003/10,508 (47.6%)
**ICD 10 code for infection n/N (%)**	70,137/83,338 (84.2%)	27,234/70,137 (38.8%)
**Antibiotic prescription n/N (%)**	37,011/83,661 (44.2%)	39,242/83,661 (46.9%)

For peer review only

Diagnosis	Number of presentations n/N (%)	Number of antibiotic prescriptions during the illness episode n/N (%)
**Respiratory**		
Common cold	34,549/53,819 (64.2)	3,643/34,549 (10.5)
Acute sinusitis	30/53,819 (0.1)	25/30 (83.3)
Acute pharyngitis	13,080/53,819 (24.3)	11,607/13,080 (88.7)
Acute tonsillitis	3,459/53,819 (6.4)	3,014/3,459 (87.1)
Other URTIs	357/53,819 (0.7)	278/357 (77.9)
Acute LRTIs	663/53,819 (1.2)	541/663 (81.6)
Chronic bronchitis, emphysema & bronchiectasis	60/53,819 (0.1)	10/60 (16.7)
Cough	1,621/53,819 (3)	99/1,621 (6.1)
Sub total	53,819 (100)	19,217/53,819 (35.7)
**Gastrointestinal**		
Bacterial intestinal infections or intoxications	199/2,706 (7.4)	127/199 (63.8)
Viral enteritis	46/2,706 (1.7)	4/46 (8.7)
GE & colitis	2,412/2,706 (89.1)	1,614/2,412 (66.9)
Appendicitis	21/2,706 (0.8)	2/21 (9.5)
Other	9/2,706 (0.3)	2/9 (22.2)
Sialoadenitis	19/2,706 (0.7)	16/19 (84.2)
Sub total	2,706 (100)	1,765/2,706 (65.2)
**Skin**		
Infective dermatitis	85/4,060 (2.1)	70/85 (82.4)
Dermatophytosis	902/4,060 (22.2)	92/902 (10.2)
Other superficial mycoses	197/4,060 (4.9)	14/197 (7.1)
Candidiasis	101/4,060 (2.5)	23/101 (22.8)
Other	64/4,060 (1.6)	52/64 (81.3)
Scabies & infestations	52/4,060 (1.3)	8/52 (15.4)
Cellulitis & abscesses	841/4,060 (20.7)	618/841 (73.5)
Bacterial skin infections	533/4,060 (13.1)	464/533 (87.1)
Furuncles, caruncles & cysts	947/4,060 (23.3)	780/947 (82.4)
Other local infection of the skin & subcutaneous tissue	338/4,060 (8.3)	290/338 (85.8)
Sub total	4,060 (100)	2,411/4060 (59.4)
**Eye**		
Conjunctivitis	2,097/2,698 (77.7)	330/2,097 (15.7)
Hordeolum & chalazion	319/2,698 (11.8)	256/319 (80.3)
Other inflammation of the eyelid & orbit	268/2,698 (9.9)	98/268 (36.6)
Trachoma	14/2,698 (0.5)	5/14 (35.7)

For peer review only

Sub total	2,698 (100)	689/2,698 (25.5)
**Ear**		
Otitis externa	464/753 (61.6)	369/464 (79.5)
Otitis media	243/753 (32.3)	197/243 (81.1)
Mastoiditis	16/753 (2.1)	9/16 (56.3)
Perforation of tympanic membrane & other disorders	30/753 (4)	25/30 (83.3)
Sub total	753 (100)	600/753 (79.7)
**Urogenital**		
Acute tubulo-interstitial nephritis	36/1,871 (1.9)	32/36 (88.9)
Other	17/1,871 (0.9)	12/17 (70.6)
Cystitis, UTI, dysuria, urethritis & urethral syndrome	1,370/1,871 (73.2)	1,291/1,370 (94.2)
Other disorders of male genital organs	32/1,871 (1.7)	20/32 (62.5)
Other inflammatory disorders of female pelvic organs	148/1,871 (7.9)	115/148 (77.7)
Other inflammatory disorders of the vagina & vulva	268/1,871 (14.3)	149/268 (55.6)
Sub total	1,871 (100)	1,619/1,871 (86.5)
**Other**		
Bacterial	85/3,208 (2.7)	28/85 (32.9)
Unknown aetiology	33/3,208 (1)	14/33 (42.4)
Viral	728/3,208 (22.7)	153/728 (21)
Fungal	36/3,208 (1.1)	2/36 (5.6)
Protozoal	10/3,208 (0.3)	0/10 (0)
Parasitic	1,880/3,208 (58.6)	99/1,880 (5.3)
Nausea & vomiting	268/3,208 (8.4)	30/268 (11.2)
Fever of unknown or other origin	168/3,208 (5.2)	10/168 (6)
Sub total	3,208 (100)	336/3,208 (10.5)

Table S4: The number of presentations per diagnosis and system and whether antibiotics were prescribed for that illness episode

For peer review only

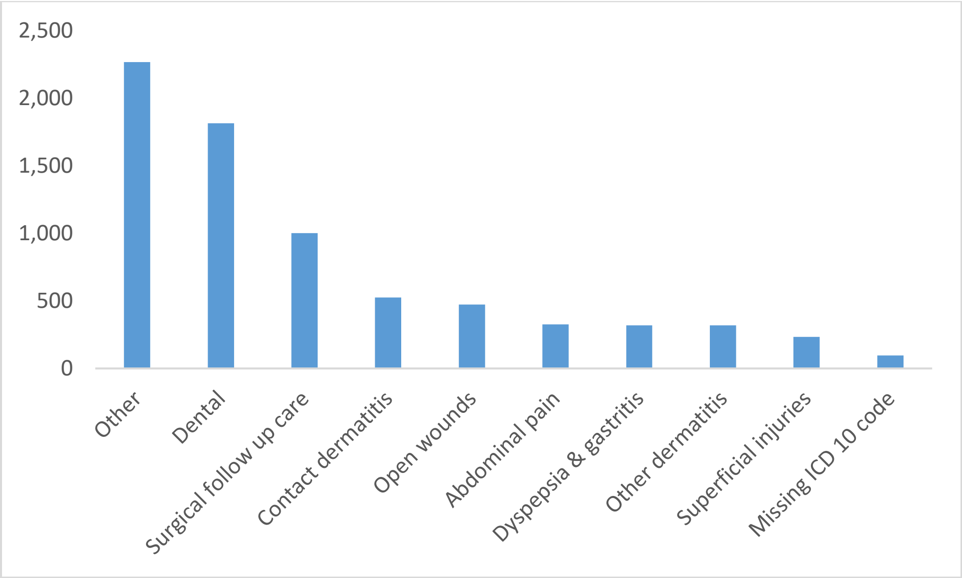

Figure S1: Single diagnoses used for antibiotic prescriptions without a history of fever, temperature or ICD 10 code for infection

For peer review only

ORD statement – checklist of items, extended from the STROBE statement, that should be reported in observational stud ollected health data.

	Item No.	STROBE items	Location in manuscript where items are reported	RECORD items	Location manuscri where ite reported
**bstract**					
	1	(a) Indicate the study's design with a commonly used term in the title or the abstract (b) Provide in the abstract an informative and balanced summary of what was done and what was found		RECORD 1.1: The type of data used should be specified in the title or abstract. When possible, the name of the databases used should be included.<br><br>RECORD 1.2: If applicable, the geographic region and timeframe within which the study took place should be reported in the title or abstract.<br><br>RECORD 1.3: If linkage between databases was conducted for the study, this should be clearly stated in the title or abstract.	Abstract p 11<br><br>Abstract p 11&13<br><br>NA
**on**					
d	2	Explain the scientific background and rationale for the investigation being reported			Page 3 Backgrou section
	3	State specific objectives, including any prespecified hypotheses			Page 3, lir Page 4, lir 52: Study outcomes
gn	4	Present key elements of study design early in the paper			Page 3 lin page 4 page 5
	5	Describe the setting, locations, and relevant dates, including periods of recruitment, exposure,			Page 3 lin page 5 lin

For peer review only

		follow-up, and data collection			
s	6	*(a) Cohort study* - Give the eligibility criteria, and the sources and methods of selection of participants. Describe methods of follow-up<br>*Case-control study* - Give the eligibility criteria, and the sources and methods of case ascertainment and control selection. Give the rationale for the choice of cases and controls<br>*Cross-sectional study* - Give the eligibility criteria, and the sources and methods of selection of participants<br><br>*(b) Cohort study* - For matched studies, give matching criteria and number of exposed and unexposed<br>*Case-control study* - For matched studies, give matching criteria and the number of controls per case		RECORD 6.1: The methods of study population selection (such as codes or algorithms used to identify subjects) should be listed in detail. If this is not possible, an explanation should be provided.<br><br>RECORD 6.2: Any validation studies of the codes or algorithms used to select the population should be referenced. If validation was conducted for this study and not published elsewhere, detailed methods and results should be provided.<br><br>RECORD 6.3: If the study involved linkage of databases, consider use of a flow diagram or other graphical display to demonstrate the data linkage process, including the number of individuals with linked data at each stage.	Page 3 lin<br>page 4 30<br>line 35<br><br>NA<br><br>NA
	7	Clearly define all outcomes, exposures, predictors, potential confounders, and effect modifiers. Give diagnostic criteria, if applicable.		RECORD 7.1: A complete list of codes and algorithms used to classify exposures, outcomes, confounders, and effect modifiers should be provided. If these cannot be reported, an explanation should be provided.	Page 5 lin<br>suppleme<br>material
es/<br>nt	8	For each variable of interest, give sources of data and details of methods of assessment (measurement).<br>Describe comparability of assessment methods if there is more than one group			Page 5 lin<br>page 6 lin

For peer review only

	9	Describe any efforts to address potential sources of bias			NA
	10	Explain how the study size was arrived at			NA
e	11	Explain how quantitative variables were handled in the analyses. If applicable, describe which groupings were chosen, and why			Page 5 lin<br>page 6 lin
	12	(a) Describe all statistical methods, including those used to control for confounding<br>(b) Describe any methods used to examine subgroups and interactions<br>(c) Explain how missing data were addressed<br>(d) *Cohort study* - If applicable, explain how loss to follow-up was addressed<br>*Case-control study* - If applicable, explain how matching of cases and controls was addressed<br>*Cross-sectional study* - If applicable, describe analytical methods taking account of sampling strategy<br>(e) Describe any sensitivity analyses			Page 5 lin<br>15, 37- pa<br>line 35
s and<br>ethods		..		RECORD 12.1: Authors should describe the extent to which the investigators had access to the database population used to create the study population.<br><br>RECORD 12.2: Authors should provide information on the data	Page 4 lin<br>page 5 lin

For peer review only

				cleaning methods used in the study.	Page 5 lin
		..		RECORD 12.3: State whether the study included person-level, institutional-level, or other data linkage across two or more databases. The methods of linkage and methods of linkage quality evaluation should be provided.	NA
s	13	(a) Report the numbers of individuals at each stage of the study (*e.g.*, numbers potentially eligible, examined for eligibility, confirmed eligible, included in the study, completing follow-up, and analysed)<br>(b) Give reasons for non-participation at each stage.<br>(c) Consider use of a flow diagram		RECORD 13.1: Describe in detail the selection of the persons included in the study (*i.e.,* study population selection) including filtering based on data quality, data availability and linkage. The selection of included persons can be described in the text and/or by means of the study flow diagram.	Page 3 lin 48, page 4 Page 6 lin page 7 lin
e data	14	(a) Give characteristics of study participants (*e.g.*, demographic, clinical, social) and information on exposures and potential confounders<br>(b) Indicate the number of participants with missing data for each variable of interest<br>(c) *Cohort study* - summarise follow-up time (*e.g.*, average and total amount)			Page 7 lin page 8 lin
ata	15	*Cohort study* - Report numbers of outcome events or summary measures over time<br>*Case-control study* - Report numbers in each exposure category, or summary measures of exposure			Page 7 lin page 8 lin

For peer review only

		Cross-sectional study - Report numbers of outcome events or summary measures			
ts	16	(a) Give unadjusted estimates and, if applicable, confounder-adjusted estimates and their precision (e.g., 95% confidence interval). Make clear which confounders were adjusted for and why they were included<br>(b) Report category boundaries when continuous variables were categorized<br>(c) If relevant, consider translating estimates of relative risk into absolute risk for a meaningful time period			Page 7 lin<br>page 8 lin
yses	17	Report other analyses done—e.g., analyses of subgroups and interactions, and sensitivity analyses			Page 8 lin<br>page 9 lin
	18	Summarise key results with reference to study objectives			Page 9 lin<br>30
s	19	Discuss limitations of the study, taking into account sources of potential bias or imprecision. Discuss both direction and magnitude of any potential bias		RECORD 19.1: Discuss the implications of using data that were not created or collected to answer the specific research question(s). Include discussion of misclassification bias, unmeasured confounding, missing data, and changing eligibility over time, as they pertain to the study being reported.	Page 10 li<br>54, page 1<br>6-14
on	20	Give a cautious overall interpretation of results considering objectives, limitations, multiplicity of analyses, results from similar			Page 9 lin<br>page 11 li

For peer review only

		studies, and other relevant evidence			
bility	21	Discuss the generalisability (external validity) of the study results			Page 10 li 44
**rmation**					
	22	Give the source of funding and the role of the funders for the present study and, if applicable, for the original study on which the present article is based			Page 11 li 44
ty of aw ng		..		RECORD 22.1: Authors should provide information on how to access any supplemental information such as the study protocol, raw data, or programming code.	Page 12 li 19

: Benchimol EI, Smeeth L, Guttmann A, Harron K, Moher D, Petersen I, Sørensen HT, von Elm E, Langan SM, the RECORD V
. The REporting of studies Conducted using Observational Routinely-collected health Data (RECORD) Statement. *PLoS Medi*

is protected under Creative Commons Attribution (CC BY) license.

For peer review only
